# An Integrated Response of *Trichodesmium erythraeum* IMS101 Growth and Photo-Physiology to Iron, CO_2_, and Light Intensity

**DOI:** 10.3389/fmicb.2018.00624

**Published:** 2018-04-10

**Authors:** Tobias G. Boatman, Kevin Oxborough, Martha Gledhill, Tracy Lawson, Richard J. Geider

**Affiliations:** ^1^School of Biological Sciences, University of Essex, Colchester, United Kingdom; ^2^Chelsea Technologies Group Ltd, West Molesey, United Kingdom; ^3^Ocean and Earth Science, National Oceanography Centre Southampton, University of Southampton, Southampton, United Kingdom; ^4^GEOMAR, Helmholtz Centre for Ocean Research, Kiel, Germany

**Keywords:** *Trichodesmium erythraeum*, Cyanobacteria, ocean acidification, CO_2_, iron limitation, light intensity, fluorescence light curves, electron transport rates

## Abstract

We have assessed how varying CO_2_ (180, 380, and 720 μatm) and growth light intensity (40 and 400 μmol photons m^−2^ s^−1^) affected *Trichodesmium erythraeum* IMS101 growth and photophysiology over free iron (Fe′) concentrations between 20 and 9,600 pM. We found significant iron dependencies of growth rate and the initial slope and maximal relative PSII electron transport rates (rP_m_). Under iron-limiting concentrations, high-light increased growth rates and rP_m_; possibly indicating a lower allocation of resources to iron-containing photosynthetic proteins. Higher CO_2_ increased growth rates across all iron concentrations, enabled growth to occur at lower Fe′ concentrations, increased rP_m_ and lowered the iron half saturation constants for growth (K_m_). We attribute these CO_2_ responses to the operation of the CCM and the ATP spent/saved for CO_2_ uptake and transport at low and high CO_2_, respectively. It seems reasonable to conclude that *T. erythraeum* IMS101 can exhibit a high degree of phenotypic plasticity in response to CO_2_, light intensity and iron-limitation. These results are important given predictions of increased dissolved CO_2_ and water column stratification (i.e., higher light exposures) over the coming decades.

## Introduction

In vast regions of the oligotrophic tropical and sub-tropical open oceans, input of new nitrogen is primarily dependent on the N_2_-fixing capabilities of diazotrophic cyanobacteria, including unicellular cyanobacteria such as UCYN-A (Martinez-Perez et al., 2016) and filamentous cyanobacteria such as *Trichodesmium* spp. (Carpenter and Capone, 1992; Capone et al., 1997; Campbell et al., 2005). *Trichodesmium* spp. are a fundamentally important organism as they represent up to 50% of new nitrogen in some regions (Karl et al., 1997; Capone et al., 2005), and contribute between 80 and 110 Tg of fixed N_2_ to the open ocean ecosystems per year (Capone et al., 1997).

Diazotrophy is performed by the two-component enzyme nitrogenase, which comprises of an iron-molybdenum protein (dinitrogen reductase) and an iron protein (nitrogenase reductase). The former reduces the latter using a low-potential electron donor (ferredoxin) and consumes two molecules of ATP per electron. Nitrogenase contains 19 iron atoms per heterodimeric protein molecule (Shi et al., 2007). This is important because iron is a cofactor for a whole range of enzymes involved in photosynthetic and respiratory electron transport, nitrate and nitrite reduction, chlorophyll synthesis and other biosynthetic or degradative reactions (Geider and La Roche, 1994). *Trichodesmium's* dependence on diazotrophy means the genus has a relatively high metalloenzyme inventory. As a result, iron availability may be critical in controlling rates of nitrogen fixation in large areas of the open ocean (Rueter, 1988; Rueter et al., 1992; Falkowski and Raven, 1997; Wu et al., 2000).

The majority of Fe(III) in the open ocean is chelated by organic compounds (Shi et al., 2010) with the remaining fraction present as hydrolysed species [Fe(OH${)}_{\text{x}}^{\left(3-\text{x}\right)+}$]. The neutral tri-hydrolysed species [Fe(OH)_3_] has very low solubility. As ocean pH decreases, so too does the hydroxide concentration, which slightly increases the solubility of iron in seawater (Liu and Millero, 1999). As hydroxide ions and organic chelators compete for the binding of Fe(III), ocean acidification will alter the organic chelation of iron, the degree of which is subject to the pK_a_ of the binding site (Shi et al., 2007). This may therefore act to limit the bioavailability of iron. This is particularly important for areas of the ocean where a significant fraction of new iron comes from dissolved iron in deep waters. In areas where the major source is particulate iron, this may be partially compensated for by an increased ability of some chelators to dissolve iron from oxyhydroxides, arial dust, and siderophores (Shi et al., 2007; Rubin et al., 2011), and/or by enhanced photo-induced redox cycling (Croot and Heller, 2012).

The exceedingly low solubility of ferric iron (Fe^3+^) (10^−18^ M at pH 7.0 and effectively insoluble at higher pH), coupled with the fact that a major source of iron flux to the open ocean gyres is from atmospheric dust deposition (Gao et al., 2001) has led cyanobacteria to employ various mechanisms to (i), increase iron acquisition from the environment (i.e., outer membrane receptor proteins, ligand complexes, periplasmic and cytoplasmic iron transport proteins) (Braun and Killmann, 1999) (ii), decrease the cellular iron requirement by regulating the expression of genes encoding for cellular iron homeostasis (i.e., IsiA, IsiB, Fur) (Webb et al., 2001) and (iii), intracellular recycling of iron (Saito et al., 2011). These mechanisms allow increased production of high-affinity iron transporters and down-regulation of membrane-bound photosynthetic electron transport (PET) components in proportion to their iron requirement (Ivanov et al., 2000). For example, the Cu-containing plastocyanin in place of cytochrome c_553_ and flavodoxin in place of ferredoxin (Geider and La Roche, 1994).

There is still a dearth of knowledge regarding *Trichodesmium's* growth and photo-physiological response to iron-limitation; especially in combination with the effects of ocean acidification (Berman-Frank et al., 2001, 2007; Shi et al., 2007, 2010). Given the significant role that *Trichodesmium* plays in biogeochemical cycles, it would be extremely useful for future climate models if such responses were better understood.

Recent studies have considered integrated responses of CO_2_ and light (Kranz et al., 2010a), CO_2_ and iron (Shi et al., 2012), CO_2_ and ${\text{NO}}_{3}^{-}$ (Eichner et al., 2014), and CO_2_, temperature and light (Boatman et al., 2017). These studies illustrate how *Trichodesmium's* productivity and growth is modulated by numerous environmental factors, highlighting the need for more systematic, multivariable experiments under co-limiting conditions. This requires that fully-acclimated balanced growth is established by culturing *Trichodesmium* for long time periods, under controlled and defined conditions.

Like the majority of experiments investigating physiological effects of iron-limitation, most research involving *Trichodesmium* has used a single independent variable (e.g., iron) whilst keeping CO_2_ and light intensity constant (Berman-Frank et al., 2001, 2007; Chappell and Webb, 2010). Most of these studies incorporate 5–8 treatments and culturing periods that may not have been long enough to establish balanced growth. In addition, growth conditions were frequently undefined.

Notable exceptions were the studies by Shi et al. (2012), who investigated iron-limitation at three CO_2_ concentrations at constant light intensity and temperature, and Hong et al. (2017), who investigated two CO_2_ concentrations at constant light intensity and temperature. Our approach comprised a systematic, multivariable experiment; where numerous *T. erythraeum* IMS101 treatments were grown over long durations with controlled and well-defined growth conditions. Our aim was to assess the response of *T. erythraeum* IMS101 growth, relative photosystem II (PSII) electron transport rates and photo-physiology to free iron (Fe′), and investigate how the integrated effect of CO_2_ and light intensity influence this response.

## Materials and methods

Prior to the experiment, *T. erythraeum* IMS101 was maintained in the exponential growth phase in semi-continuous cultures at two light intensities (40 and 400 μmol photons m^−2^ s^−1^) on a 12:12 light:dark cycle at three targeted CO_2_ concentrations (180, 380, and 720 μatm) and optimal growth temperature (26°C ± 0.2) for a period of ~10 months. Each culture was used to inoculate *T. erythraeum* IMS101 across a range (20–13,000 pM) of Fe′ concentrations (4 treatments at LL and 5 treatments at HL), generating 27 experimental treatments in total. All 27 treatments were maintained in exponential growth phase in semi-continuous cultures at the specific growth conditions (i.e., light intensity, temperature, CO_2_ and Fe′) for ~6 months (~14 generations for the slowest growing cultures and 95 generations for the faster growing cultures) to ensure fully acclimated balanced growth was achieved.

### Experimental setup

Cultures were grown at low volume (5 mL) in 12 mL polypropylene (PP) screw cap test tubes, incubated in a custom-made, water-jacketed aluminium temperature block illuminated from below. Sampling methodology and analytical techniques followed those described in Boatman et al. (2017); and involved median growth rates being determined from a minimum of three replicate growth curves. Experimental setup was slightly different as we employed trace metal-clean culturing techniques and used a modified YBCII growth medium, polypropylene growth tubes, and a modified approach to target a CO_2_ concentration as described below.

Growth rates were quantified from the linear regression of the slope of *ln* minimum fluorescence (*F*_*o*_) measured daily (between 09:00 to 10:30) on dark-acclimated cultures (20 min) using a FRRfII FastAct Fluorometer system (Chelsea Technologies Group Ltd, UK). Cultures were kept within the early part of the exponential growth phase and optically thin to avoid nutrient limitation and self-shading as well as to minimise CO_2_ drift, as described in Boatman et al. (2017).

### Culture medium preparation

Single batches (4 L each) of filter-sterilised (0.25 μm pore) YBCII media (Chen et al., 1996) were made at each total iron (Fe_T_) concentration (i.e., 400, 200, 100, 40, 4 nM) in acid washed plastic containers. Hydrated and anhydrous salts were added with Milli-Q water (Millipore Milli-Q Biocel, ZMQS60FOI) and the pH adjusted to ~8.2 using filter-sterilised NaOH (pH checked by taking 5 mL aliquots). Trace metal contaminants were removed by filtering through a Chelex column setup within a trace metal-clean laminar flow cabinet (Class II). Trace metals and f/2 vitamins were added to each 4 L container using a revised EDTA concentration (20 μM) (Shi et al., 2012). Varying amounts of a 40 μM Fe stock (FeCl_3_:Na_2_EDTA) were added to give the range of Fe_T_ concentrations. Each container was filter-sterilised (0.2 μm pore) into 1 L (sterile) plastic stericups (no headspace) (Fisher Scientific 10518822, UK), and stored within double zip-locked polyethylene (PE) bags. Prior to use, growth tubes were acid washed (2 weeks in 10% HCl), rinsed with Milli-Q water (Millipore Milli-Q Biocel, ZMQS60FOI), microwave sterilised, air dried in a laminar flow cabinet (Class II), and stored within double zip-locked PE bags (Anderson and Morel, 1982). Each dilution was made into a new tube to avoid the build-up of contaminants.

### Calculating iron speciation

The speciation program visual MINTEQ (Gustafsson, 2012) was used to calculate the solubility and organic complexation of iron, as well as determine the chemical speciation as a function of pH (Gledhill et al., 2015). Modelled concentrations (M) of Fe_2_(OH)_2_(EDTA${)}_{2}^{-4}$, Fe(EDTA)^−^, FeH(EDTA) (aq) and FeOH(EDTA)^−2^ were summed and used in the calculation of the photo-redox disassociation of Fe(EDTA), which made up the dominant source of Fe′ in the media. The photo-redox calculation was based upon a set of rate constant equations defined by Sunda et al. (2005), giving a diurnally averaged Fe′ concentration in the growth media (Supplementary Table 1);

(1) $$\begin{array}{r} {Fe\left(III\right)}^{\prime }=\frac{\left[Fe\left(EDTA\right)\;\cdot \;\left({K_{d}}^{\prime }\left(dark\right)\;+\;E_{hv}\;\cdot \;k_{hv}\;\cdot \;\left(\frac{LP}{24}\right)\right)\right]}{\left[EDTA^{*}\right]} \end{array}$$

(2) $$\begin{array}{r} {K_{d}}^{\prime }\left(dark\right)=10^{\left(2.427\;\cdot \;\left[pH\right]\;\cdot \;-26.84\right)} \end{array}$$

(3) $$\begin{array}{r} K_{hv}=10^{\left(0.776\;\cdot \;\left[pH\right]\;\cdot \;-12.92\right)} \end{array}$$

where Fe(III)′ is the free iron (Fe′) concentration (M); Fe(EDTA) is the total iron (Fe_T_) concentration (M); [EDTA^*^] is the total EDTA concentration (M); K_d_′ (dark) is the constant in the dark (= *k*_*d*_*/k*_*f*_), which is the ratio of the rate constants for the disassociation and formation of Fe(EDTA) chelates; K_hv_ is the rate constant (= *k*_*hv*_*/k*_*f*_) for Fe(EDTA) photolysis at a specific light intensity; E_hv_ is the light intensity (μmol photons m^−2^ s^−1^) relative to that at which K_hv_ was measured (i.e., 500 μmol photons m^−2^ s^−1^); LP is the light period of the culture treatment (h^−1^), and pH was a post-culturing measurement on the NBS scale.

### Inorganic carbon chemistry

The inorganic carbon chemistry (Ci) of each media bottle was determined from a 15 mL sample for total dissolved inorganic carbon (TIC) analysis (Shimadzu TOC-V Analyser & ASI-V Autosampler), and a 10 mL sample for pH (Thermo Scientific Orion Ross Ultra pH Electrode EW-05718-75, UK). All carbon chemistry calculations were made in *CO2SYS* (Lewis and Wallace, 1998), using the 1st and 2nd equilibrium constants (K1 and K2) for carbonic acid (Millero, 2010), the dissociation constant for KSO_4_ (Dickson, 1990), the boric acid constant (KB) (Lee et al., 2010), and the total pH scale. The pH probes were rinsed and calibrated with fresh (<2 weeks) artificial seawater buffers (TRIS and AMP) prior to use (Dickson, 1993).

Once a culture reached a pre-determined *F*_*o*_, it was diluted (0.5 mL culture to 4.5 mL media) with filter-sterilised (0.2 μm pore) YBCII media to return the culture to a starting *F*_*o*_ value. To obtain a target CO_2_ concentration in the YBCII media, medium was bubbled with a CO_2_-air mixture (BOC Industrial Gases, UK) using an acid washed (10% HCl), microwave sterilised section of PTFE tubing. A series of 5 ml aliquots were taken to measure the pH (precision ± 0.002) until the target pH (and thus the target CO_2_) had been achieved. Once at the target CO_2_ concentration (±1%), the medium was immediately distributed into the test tubes, already containing the 0.5 mL of culture. The gaseous headspace was flushed with a filtered (0.2 μm pore) standard gas mixture at the target CO_2_ concentration (BOC Industrial Gases, UK) and the screw caps tightened. Parafilm was wrapped around the caps before the tubes were removed from the laminar flow cabinet.

Prior to every dilution, a 2 mL sample of culture and a 2 mL sample of filtrate were collected within 5 mL plastic cryogenic vials (Sigma-Aldrich V5257-250EA). Filtrate was used to measure the post-culturing pH. Assuming a constant alkalinity throughout the entire growth phase (Kranz et al., 2010b), the post-culturing CO_2_ was calculated from the post-culturing pH and initial alkalinity. The second 2 mL aliquot of culture was pipetted into a new growth tube to run a fluorescence light curve (FLC).

### Fluorescence light curves

Triplicate FLCs were performed for each treatment using an FRRfII FastAct Fluorometer system (Chelsea Technologies Group Ltd, UK) on dark-acclimated cultures (20 min). The FLCs lasted ~1 h and consisted of 12 light steps ranging between 6 and 1,400 μmol photons m^−2^ s^−1^, each lasting 5 min in duration.

The operating efficiency of PSII (*F*_*q*_′/*F*_*m*_′) was calculated as follows;

(4) $$\frac{F_{q}\prime }{F_{m}\prime }=\left[\frac{F_{m}\prime -F^{\prime }}{F_{m}\prime }\right]$$

where *F*_*m*_′ is the maximum fluorescence in the light-acclimated state and *F*′ is the steady-state fluorescence at any point.

Baseline fluorescence (*F*_*b*_) originating from sources other than functional, photosynthetically active PSII was determined using the following equation (Oxborough, 2012);

(5) $$\begin{array}{r} F_{b}=F_{m}-\left(\frac{F_{v}}{{F_{v}/F_{m}}^{*}}\right) \end{array}$$

where *F*_*m*_ is the maximum fluorescence in the dark-acclimated state, *F*_*v*_ (= *F*_*m*_ − *F*_*o*_) is the variable fluorescence in the dark-acclimated state and *F*_*v*_*/F*_m_^*^ is the assumed *F*_*v*_*/F*_*m*_ from functional PSII. In this study, the value of *F*_*v*_*/F*_m_^*^ was set to the highest measured value from all treatments under iron-replete conditions. This method assumes that across environmental gradients, all photosynthetically active PSII operate with the same intrinsic photochemical efficiency in the dark-acclimated state. The impact of free phycobilisome complexes within the cell is assumed negligible, as their peak emission wavelengths are too short for detection by the FRRfII.

When applicable (i.e., *F*_*v*_*/F*_*m*_ < *F*_*v*_*/F*_m_^*^), *F*_*b*_ was subtracted from *F*′ and *F*_*m*_′, and *F*_*q*_′/*F*_*m*_′ recalculated.

Relative PSII electron transport rates (rP) were calculated as follows;

(6) $$\text{rP}=\left(\frac{F_{q}\prime }{F_{m}\prime }\right)\;\cdot \;E$$

where *F*_*q*_′*/F*_*m*_′ is the operating efficiency of PSII (baseline corrected if required) and E is the actinic light intensity (μmol photons m^−2^ s^−1^).

### Curve fitting of growth rate data

Additional growth rate and FLC data points were incorporated from the temperature and light response curves reported in Boatman et al. (2017). These experiments shared identical growth light intensities (low light = 40 μmol photons m^−2^ s^−1^, high light = 400 μmol photons m^−2^ s^−1^), light:dark cycle (12:12), CO_2_ concentrations (low-CO_2_ = 180 μatm, mid-CO_2_ = 380 μatm and high-CO_2_ = 720 μatm), and growth temperature (26°C); only differing in the use of non-chelated hydrated and anhydrous salts as well as the YBCII EDTA concentration (i.e., 2 μM).

Growth rate-Fe (μ-Fe) curves were modelled using a Michaelis-Menten equation (Michaelis and Menten, 1913);

(7) $$\begin{array}{r} \mu =\;\left[\frac{\mu_{m}\cdot \text{F}{\text{e}}^{\text{′}}}{\left({\text{K}}_{\text{m}}\;+\;{\text{Fe}}^{\prime }\right)}\right] \end{array}$$

where μ_m_ is the maximum growth rate (d^−1^); Fe′ is the free Fe concentration of the media (pM) and K_m_ is the half saturation concentration (pM).

Curve fitting was performed on the median growth rate for each CO_2_ and light treatment, using a non-linear least squares algorithm to produce curves of best fit (*r*^2^ > 0.817). Statistical analysis was performed using *F*-tests; analysing the variance of separate and combined CO_2_ curve fits by comparing a calculated *F*-statistic to an *F*-value at a 0.05 alpha level.

### Curve fitting of FLC data

Relative electron transport rates (rP) were modelled using a P-E equation (Platt and Jassby, 1976), and were performed on each replicate using a Marquardt–Levenberg least squares algorithm to generate the best fit (*r*^2^ > 0.993);

(8) $$\text{rP}={\text{rP}}_{\text{m}}\prime \cdot \;\left[1-e\left(\frac{-\alpha \;\cdot \;E}{{\text{rP}}_{\text{m}}\prime }\right)e\left(\frac{-\beta \;\cdot \;E}{{\text{rP}}_{\text{m}}\prime }\right)\right]$$

where rP_m_′ is the maximum relative PSII electron transport rate (unitless); α is the initial slope of the rP-light curve (dimensionless); β is the parameter that accounts for downregulation and/or photoinhibition at supra-optimal light intensities (dimensionless); and E is the light intensity (μmol photons m^−2^ s^−1^).

The achieved maximum relative PSII electron transport rate (rP_m_), light intensity at which rP was maximal (E_opt_) and light-saturation parameter (E_k_) were calculated from the fitted parameters as follows:

(9) $$\begin{array}{r} {\text{rP}}_{m}=\;{\text{r}{\text{P}}_{\text{m}}}^{\prime }\cdot \left(\frac{\alpha }{\alpha +\beta }\right)\cdot {\left(\frac{\beta }{\alpha +\beta }\right)}^{\frac{\beta }{\alpha }} \end{array}$$

(10) $$E_{opt}=\frac{{\text{rP}}_{\text{m}}\prime }{\alpha }\cdot ln\left(\frac{\alpha +\beta }{\beta }\right)$$

(11) $$\begin{array}{r} E_{k}=\;\frac{\text{r}{\text{P}}_{\text{m}}}{\alpha } \end{array}$$

## Results

Overall, CO_2_ concentrations in the cultures at the time of sampling were between 55 and 75 μatm lower than the target *p*CO_2_ concentrations (i.e., 180, 380 and 720 μatm). This was due to drawdown of TIC associated with biomass production and was similar across all iron and light treatments (Table 1).

**Table 1:** The growth conditions (± SE) of *T. erythraeum* IMS101 cultures.

**Variables**	**Units**	**Low CO**_**2**_		**Mid CO**_**2**_		**High CO**_**2**_	
		**LL**	**HL**	**LL**	**HL**	**LL**	**HL**
pH	Total	8.426	8.426	8.145	8.135	7.853	7.887
H^+^	nM	3.8 (0.2)	3.8 (0.1)	7.2 (0.1)	7.3 (0.1)	14.0 (0.4)	13.0 (0.4)
A_T_	μM	2,396 (155)	2,399 (77)	2,453 (82)	2,324 (41)	2,256 (54)	2,427 (87)
TIC	μM	1,740 (106)	1,751 (54)	2,009 (66)	1,906 (35)	2,001 (45)	2,138 (72)
${\text{HCO}}_{3}^{-}$	μM	1,326 (66)	1,344 (36)	1,729 (53)	1,647 (30)	1,835 (39)	1,949 (62)
${\text{CO}}_{3}^{2-}$	μM	410 (41)	403 (19)	271 (13)	250 (5)	147 (7)	171 (10)
CO_2_	μM	3.4 (0.1)	3.5 (0.1)	8.6 (0.2)	8.4 (0.2)	17.8 (0.3)	17.4 (0.1)
*p*CO_2_	μatm	126 (4)	129 (2)	318 (6)	312 (7)	662 (12)	644 (3)
Chl *a*	μg L^−1^	12.3 (1.8)	17.9 (3.1)	19.9 (3.1)	35.0 (5.5)	21.4 (3.3)	33.7 (6.7)
*n*		11	14	17	30	15	15

*Cultures were acclimated to three target CO_2_ concentrations (Low CO_2_ = 180 μatm, Mid CO_2_ = 380 μatm and High CO_2_ = 720 μatm), saturating light intensity (400 μmol photons m^−2^ s^−1^), optimal temperature (26°C), across a range of Fe′ concentrations (~20–1,010 pM). Iron treatments are grouped together to present the growth conditions for each CO_2_ and light treatment. The pH was measured prior to every dilution, while the bicarbonate (${\text{HCO}}_{\text{3}}^{-}$), carbonate (${\text{CO}}_{\text{3}}^{\text{2}-}$), CO_2_, and pCO_2_ concentrations were calculated via CO2SYS using the post-culturing measurements of pH and the initial alkalinity (A_T_) concentration. Note that the individual pH-values were converted to a H^+^ concentration, allowing a mean pH value to be calculated*.

### Iron limited response of growth rate and photophysiology

**Figure 1:**
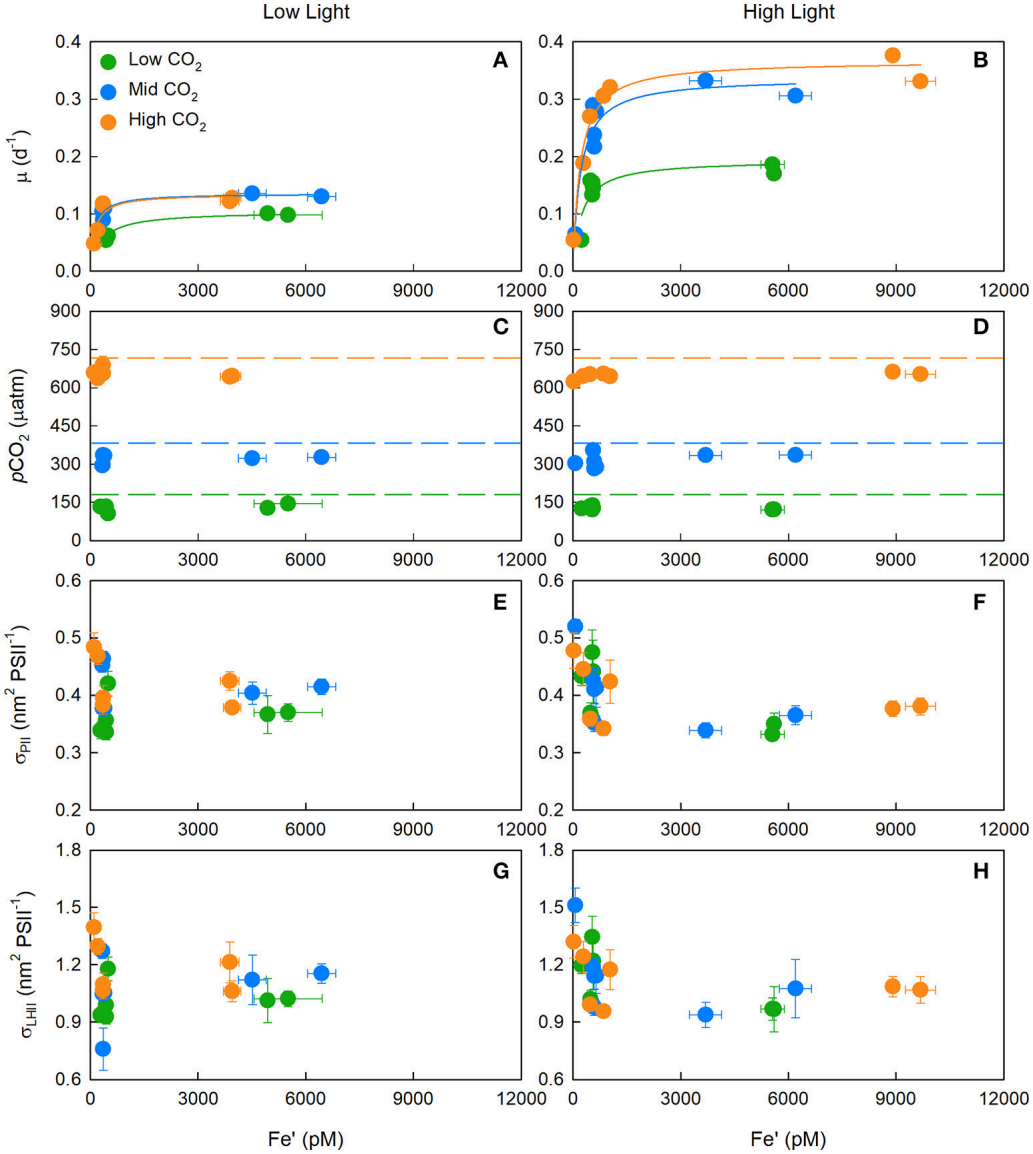
The median (± SE) growth rate (μ) **(A,B)**, and mean (± SE) *p*CO_2_ **(C,D)**, absorption cross section of PSII photochemistry (σ_PII_) **(E,F)**, and absorption cross section of PSII light harvesting (σ_LHII_ = σ_PII_/*F*_*v*_/*F*_*m*_**) (G,H)** for *T. erythraeum* IMS101. Prior to calculating σ_LHII_, the photochemical efficiency of PSII in the dark-acclimated state (*F*_*v*_/*F*_*m*_) was corrected for baseline fluorescence (*F*_*b*_) by assuming *F*_*v*_*/F*_m_^*^ = 0.362. Left-hand panels **(A,C,E,G)** are low light treatments while right-hand panels **(B,D,F,H)** are high light treatments. Cultures were acclimated to three targeted CO_2_ concentrations [Low = 180 μatm (green circles), Mid = 380 μatm (blue circles) and High = 720 μatm (orange circles)], two light intensities (LL = 40 μmol photons m^−2^ s^−1^, HL = 400 μmol photons m^−2^ s^−1^), across a range of Fe′ concentrations (~20–9,600 pM) and at optimal temperature (26°C).

Growth rates decreased significantly with a decrease in Fe′ (Figures 1A,B). Acclimation to high CO_2_ enabled growth to occur at comparatively lower Fe′ concentrations at both low and high light (Figures 1A,B). Maximum (iron-replete) growth rates (μ_m_) exhibited a CO_2_ response; where at low light, μ_m_ increased significantly by 30% from low to mid CO_2_ [*F*_(2, 8)_ = 14.00, *p* < 0.05], and 29% from low to high CO_2_ [*F*_(2, 8)_ = 6.00, *p* < 0.05]. At high light, μ_m_ increased significantly by 74% from low to mid CO_2_ [*F*_(2, 10)_ = 26.25, *p* < 0.05], and 90% from low to high CO_2_ [*F*_(2, 10)_ = 51.17, *p* < 0.05; Table 2]. There were no significant differences in μ_m_ between mid and high CO_2_ treatments at low [*F*_(2, 8)_ = 0.01, *p* > 0.05] or high light [*F*_(2, 10)_ = 1.25, *p* > 0.05; Supplementary Table 2].

**Table 2:** The iron dependence of *T. erythraeum* IMS101 growth.

**Parameters**	**Units**	**Low CO_2_**	**Mid CO_2_**	**High CO_2_**
**LOW LIGHT**				
μ_m_	d^−1^	0.104 (0.005)^[A]^^*^	0.136 (0.006)^[B]^^*^	0.134 (0.012)^[B]^^*^
K_m_	pM	307 (56)^[A]^	116 (29)^[B]^	124 (48)^[B]^
Affinity	d^−1^ (nM)^−1^	0.339 (0.028)^[A]^^*^	1.165 (0.125)^[B]^^*^	1.083 (0.259)^[B]^^*^
**HIGH LIGHT**				
μ_m_	d^−1^	0.193 (0.021)^[A]^^*^	0.337 (0.025)^[B]^^*^	0.367 (0.016)^[B]^^*^
K_m_	pM	236 (104)	206 (72)	201 (46)
Affinity	d^−1^ (nM)^−1^	0.819 (0.248)^*^	1.639 (0.321)^*^	1.823 (0.175)^*^

*T. erythraeum IMS101 was acclimated to three target CO_2_ concentrations (Low CO_2_ = 180 μatm, Mid CO_2_ = 380 μatm, and High CO_2_ = 720 μatm), saturating light intensity (400 μmol photons m^−2^ s^−1^), optimal temperature (26°C), across a range of Fe′ concentrations (~ 20–9,600 pM). Median Fe-dependent growth rate curves were fitted using a Michaelis-Menten function, defining parameter values (± SE) for μ_m_ (d^−1^), the maximum growth rate; K_m_ (pM Fe′), the half saturation concentration and affinity [d^−1^ (nM Fe′)^−1^], the initial slope of the growth- Fe′ curve. At low light, there were 6 growth rate data points per CO_2_ treatment with two estimated parameters (df = 4); and at high light, there were 7 growth rate data points per CO_2_ treatment with two estimated parameters (df = 5). Letters in parenthesis indicate significant differences between CO_2_ treatments (F-test, P < 0.05); where [B] is significantly greater than [A]. An asterisk indicates a significant difference between light treatments*.

At low light, increasing from low to mid CO_2_ caused a significant three-fold decrease in the half saturation concentration (K_m_) for growth [*F*_(2, 8)_ = 14.979, *p* < 0.05], as well as a three-fold increase in the affinity (initial slope) of the growth-Fe′ curve [*F*_(2, 8)_ = 11.222, *p* < 0.05]. Increasing from mid to high CO_2_ did not cause significant differences to either K_m_ [*F*_(2, 8)_ = 0.144, *p* > 0.05] or affinity [*F*_(2, 8)_ = 1.031, *p* > 0.05]. At high light, the variability in K_m_ [*F*_(4, 15)_ = 0.083, *p* > 0.05] and affinity [*F*_(2, 8)_ = 0.056, *p* > 0.05] between CO_2_ treatments was not significant. Due to the associated standard errors, there were no significance differences in K_m_ between low and high light treatments at the low [*F*_(4, 9)_ = 0.441, *p* > 0.05], mid [*F*_(4, 9)_ = 2.049, *p* > 0.05] or high [*F*_(4, 9)_ = 1.158, *p* > 0.05] CO_2_ treatments (Supplementary Tables 3–5).

For both low [*F*_(4, 12)_ = 0.333, *p* > 0.05] and high light [*F*_(4, 15)_ = 1.429, *p* > 0.05] treatments, combining the mid and high CO_2_ growth rate data did not cause a significant difference in curve fit parametrisations. However, incorporating the low CO_2_ data into a combined fit (i.e., low + mid + high CO_2_ growth rate data) caused the combined curve fit parameterisations to be significantly different to the separate CO_2_ growth rate-Fe′ curves for both low [*F*_(4, 12)_ = 14.143, *p* < 0.05] and high treatments [*F*_(4, 12)_ = 27.743, *p* < 0.05; Supplementary Table 2].

**Figure 2:**
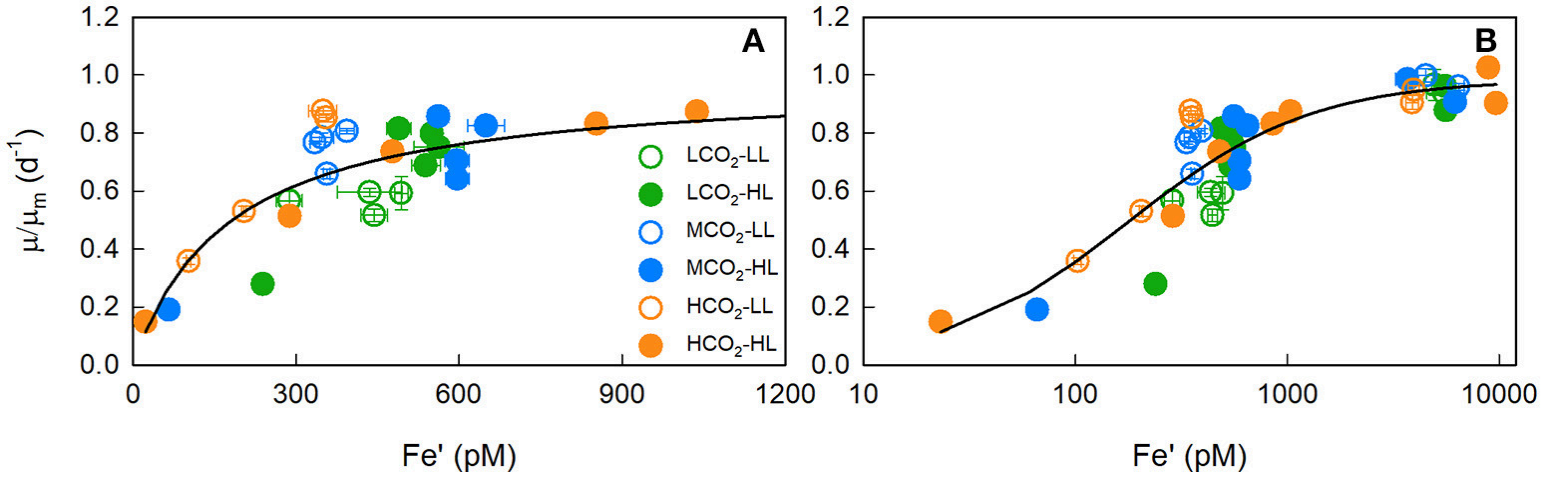
The iron dependency of maximum-normalised growth rates (μ/μ_m_). Panel **(A)** shows the iron-limitation range (0–1,200 pM) plot on a linear scale while panel **(B)** shows the full range (0–12 nM) of data plot on a log scale, and includes the data points from Boatman et al. (2017). *T. erythraeum* IMS101 was cultured at three targeted CO_2_ concentrations [Low = 180 μatm (green circles), Mid = 380 μatm (blue circles) and High = 720 μatm (orange circles)], two light intensities [LL = 40 μmol photons m^−2^ s^−1^ (open circles), HL = 400 μmol photons m^−2^ s^−1^ (closed circles)], across a range of Fe′ concentrations (~20–1,010 pM), at optimal temperature (26°C). The solid line is μ/μ_m_ modelled by a Michaelis-Menten function (K_m_ of 177 pM with an *r*^2^ of 0.803).

Growth-Fe′ curves were normalised to the modelled maximum growth rate (μ_m_) at each CO_2_ and light treatment to generate a single μ/μ_m_-Fe′ curve. Low CO_2_ data deviated either side of the modelled curve fit (Figure 2A), highlighting that the significant differences in the growth rate-Fe′ curve fits arise from differences in the growth response to iron-limitation rather than the maximal rate achieved under iron-replete conditions.

Dark-acclimated absorption cross-sections of PSII photochemistry (σ_PII_) showed little variation between CO_2_ or light treatments, but did significantly increase from ~0.4 nm^2^ PSII^−1^ under iron-replete concentrations to ~0.45 nm^2^ PSII^−1^ under iron-limited concentrations (One-Way ANOVA on Ranks, Dunn's *post-hoc*; *p* < 0.05) (Figures 1E,F). The non-baseline (*F*_*b*_) corrected photochemical efficiency of PSII (*F*_*v*_/*F*_*m*_) exhibited a strong negative correlation to the non-*F*_*b*_ corrected absorption cross section of PSII light harvesting (i.e., σ_LHII_ = σ_PII_/*F*_*v*_/*F*_*m*_) (*r*^2^ = −0.871, *p* < 0.05) (Supplementary Figure 1).

The highest *F*_*v*_/*F*_*m*_ achieved from iron-replete cultures (~0.362) was used in the calculation of *F*_*b*_ (i.e., *F*_*v*_*/F*_m_^*^). The σ_LHII_, calculated from *F*_*b*_ corrected *F*_*v*_/*F*_*m*_ values, showed no significant difference between CO_2_ or light treatments, but did significantly increase from ~1.0 nm^2^ PSII^−1^ under iron-replete conditions to >1.4 nm^2^ PSII^−1^ under iron-limiting concentrations (One-Way ANOVA on Ranks, Dunn's *post-hoc*; *p* < 0.05) (Figures 1G,H).

### Iron limited response of relative PSII electron transport rates and PSII photochemical efficiency

**Figure 3:**
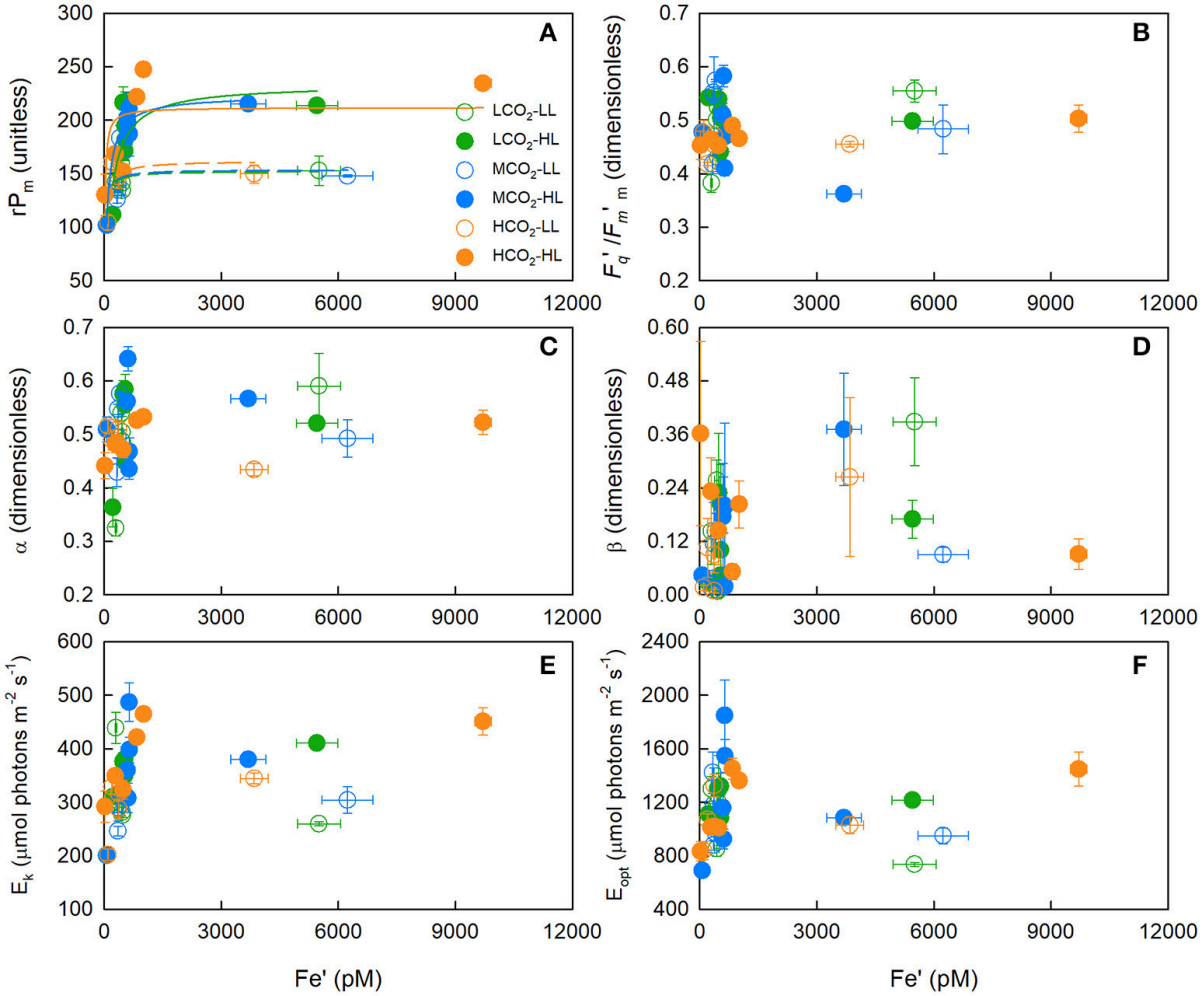
The mean (± SE) maximum relative PSII electron transport rate (rP_m_) **(A)**, photochemical efficiency of PSII in the dark-acclimated state (*F*_*v*_/*F*_*m*_) **(B)**, initial slope of the rP-Fe′ curve (α) **(C)**, slope of photoinhibition (β) **(D)**, light-saturated parameter (E_k_) **(E)**, and light intensity at which rP was maximal (E_opt_) **(F)** for *T. erythraeum* IMS101. Cultures were acclimated to three targeted CO_2_ concentrations [Low = 180 μatm (green circles), Mid = 380 μatm (blue circles) and High = 720 μatm (orange circles)], two light intensities [LL = 40 μmol photons m^−2^ s^−1^ (open circles), HL = 400 μmol photons m^−2^ s^−1^ (closed circles)], across a range of Fe′ concentrations (~20–9,600 pM), at optimal temperature (26°C). In **(A)**, the dashed and solid lines are the Michaelis-Menten function curve fits for the low light and high light treatments, respectively; where green, blue and orange lines are for low, mid and high CO_2_ treatments, respectively. Data in the iron-limited region only (20–1,010 pM Fe′) is presented in Supplementary Figure 2.

The highest maximum relative PSII electron transport rates (rP_m_) remained relatively constant down to an Fe′ concentration of ~1,000 pM (Figure 3A), and then decreased linearly with decreasing Fe′ [*t*_(2, 25)_ = 8.535, *p* < 0.0001 [rP_m_ = Fe′ · 0.1455 + 100.53, *r*^2^ = 0.732]]; where rates declined by ~ 55% as concentrations decreased from ~1,000 to 20 pM Fe′ (Supplementary Figure 2). Whilst Fe′ concentrations were dissimilar between treatments, a CO_2_ response at low and high light was evident under iron-limited conditions; where acclimation to higher CO_2_ concentrations (Fe′ < 300 pM) resulted in a comparatively higher rP_m_, α and E_k_ (Figures 3A–C). For example, at the lowest Fe′ treatments, rP_m_ was only 8% lower and 18% higher at mid and high CO_2_ relative to low CO_2_, despite Fe′ being 70 and 90% lower, respectively.

Under iron-replete conditions, CO_2_ did not affect the rP_m_ (One-Way ANOVA, Tukey *post-hoc*; *p* > 0.05) or half saturation concentration for rP_m_ (${\text{K}}_{\text{m}}^{\text{rPm}}$) at low [*F*_(3, 13)_ = 0.063, *p* > 0.05] or high light [*F*_(3, 13)_ = 2.860, *p* > 0.05; Supplementary Tables 6, 7]. Although there was a significant difference between light treatments, where low light iron-replete cultures exhibited a rP_m_ ~ 150 and high light cultures ~220 (unitless) (One-Way ANOVA, Tukey *post-hoc*; *p* < 0.05).

There were significant positive correlations between rP_m_ and the light-saturation parameter (E_k_) (*r*^2^ = 0.797, *p* < 0.05), rP_m_ and the light intensity at which rP was maximal (E_opt_) (*r*^2^ = 0.501, *p* < 0.05), and rP_m_ and the initial slopes of rP-Fe′ curves (α) (*r*^2^ = 0.435, *p* < 0.05) (Figures 3E,F). In contrast, the photoinhibition slopes (β) were not correlated to CO_2_, light or Fe′ concentration (*r*^2^ < 0.5, *p* > 0.05) (Figure 3D).

The highest light-acclimated, baseline-corrected operating efficiency of PSII (*F*_*q*_′/*F*_*m*′m_) was not correlated to Fe′ (*r*^2^ = 0.036, *p* > 0.05) (Figure 3B). Although there was a significant CO_2_ response on the operating efficiency of PSII (*F*_*q*_′/*F*_*m*_′) at the highest actinic light intensity (i.e., 1400 μmol photons m^−2^ s^−1^) for low and high light cultures, where acclimation to higher CO_2_ concentrations under iron-limited conditions resulted in a comparatively higher *F*_*q*_′/*F*_*m*_′ value (Figures 3A–C). This reflects the trends reported for rP_m_, which is to be expected given rP_m_ is a product of *F*_*q*_′/*F*_*m*_′ and actinic light intensity.

## Discussion

### Iron, CO_2_, and light dependencies on balanced growth rates

Accounting for differences in growth conditions (i.e., light intensity, CO_2_ etc.), iron-replete growth rates were similar to the majority of previous research, exhibiting similar responses under comparable Fe′ concentrations (Berman-Frank et al., 2001, 2007). Our findings show a significant CO_2_ response, affirming the observation that elevated CO_2_ produces higher growth rates, with the magnitude of the CO_2_ response increasing more under iron-replete conditions. In contrast, Shi et al. (2012) reported a decrease in growth rate at elevated CO_2_ under iron-replete conditions, which they attribute to a direct pH-mediated change in Fe′, that in turn lowers the iron uptake rate. While we cannot offer a definitive explanation for this discrepancy, it may arise from differences in culturing techniques. Specific differences could include culture medium (YBCII vs. Gulf Stream seawater), the method used to manipulate the inorganic carbon chemistry (bubbling vs. HCl/NaOH additions) and the acclimated state of the cultures; where balanced growth is only achieved after many generations (Boatman et al., 2017).

A recent study by Hong et al. (2017) reported that the positive effect of ocean acidification was due to a pH induced shift of the NH_3_/${\text{NH}}_{4}^{+}$ equilibrium, where NH_3_ concentrations in the medium declines at lower pH (i.e., higher CO_2_). Dissolved inorganic N concentrations were measured in parallel cultures (Supplementary Table 8), and for all CO_2_ and light treatments were undetectable at the start of culturing and approximately 0.8 μM post-culturing. Similar concentrations have been reported in previous studies using standard YBCII media (Mulholland and Capone, 2001; Mulholland et al., 2004; Mulholland and Bernhardt, 2005), and likely indicates some cellular leakage of ${\text{NH}}_{4}^{+}$ from cells rather than contamination of the growth medium. In any case, the ${\text{NH}}_{4}^{+}$ concentrations we measured are sufficiently low (<10 μM) to have a negligible effect on nitrogenase activity and cellular metabolism (Mulholland et al., 2001), and will not be toxic to *Trichodesmium* cells (Hong et al., 2017).

Growth rates from our highly-buffered 20 μM EDTA cultures started saturating at ~1 nM Fe′, and were comparable to the iron-replete maximum growth rates reported by Boatman et al. (2017), which used standard YBCII EDTA concentrations (2 μM). In addition, *F*_*v*_/*F*_*m*_ of the 2 μM and 20 μM EDTA, iron-replete cultures were comparable (Supplementary Figure 1). This suggests that cultures from both experiments were not affected by trace metal toxicity, as one would expect a decrease in growth and/or *F*_*v*_/*F*_*m*_ during exposure to toxic Cu^2+^ concentrations, as the primary reactions of photosynthetic will become inhibited (Cid et al., 1995; Yruela et al., 1996; Nielsen et al., 2003).

Under iron-limitation there were no differences in the dark-acclimated absorption cross-sections of PSII photochemistry (σ_PII_) or maximum photochemical efficiency of PSII (*F*_*v*_/*F*_*m*_) between low and high light treatments. Therefore, the lower initial slopes (Affinity) of the low light growth-Fe′ curves are likely due to a decrease in cellular chlorophyll *a*. Decreasing the chlorophyll a concentration conserves energy, alters the stoichiometry of iron containing components in the PET chain (Falkowski and LaRoche, 1991), and may be of importance during iron-limited, low light conditions; where a ten-fold decrease in light intensity (500 to 50 μmol photons m^−2^ s^−1^) can cause a four-fold increase in the cellular iron requirement of marine phytoplankton (Sunda and Huntsman, 1997). The combination of iron-limitation and low light conditions can significantly decrease iron uptake rates (Shi et al., 2012), creating a situation where *Trichodesmium's* cellular requirements for growth cannot be supported. Thus, when the light intensity is less than the E_k_ for *Trichodesmium* growth [~80 μmol photons m^−2^ s^−1^ at low CO_2_ and ~130 μmol photons m^−2^ s^−1^ at mid and high CO_2_ (Boatman et al., 2017)], productivity may well be constrained by a co-limitation of light and iron.

This low light mediated response also exhibited a CO_2_ dependency where (i), elevated CO_2_ enabled growth rates to occur at significantly lower Fe′ concentrations (ii), increased CO_2_ yielded higher growth rates (Figure 2) and (iii), acclimation to low CO_2_ yielded the lowest initial slope of the growth-Fe′ response (Affinity) as well as the highest iron saturation parameter (K_m_). We attribute these CO_2_ responses directly to the operation of the CCM and the ATP spent/saved for CO_2_ uptake and transport at low and high CO_2_, respectively. At high light, *Trichodesmium* likely has a lowered iron quota; therefore, whilst under iron-limitation, a low CO_2_ concentration will limit growth rates less than low light. Based on the relative growth rate curve fits (μ/μ_m_) and the *F*-test results (Supplementary Tables 2–5), we suggest that at limiting and saturating light intensities, *Trichodesmium's* present (i.e., mid CO_2_) or future (i.e., high CO_2_) μ-Fe′ response can be defined using shared model parameterisations.

### Resource allocation under iron limitation

*T. erythraeum* IMS101 exhibited an array of responses to CO_2_, light and iron-limiting conditions, indicating a high degree of phenotypic plasticity. Whilst not measured here, it is well-documented that under iron-limitation, *T. erythraeum* IMS101 downregulates the genes that encode for major iron-binding proteins. For example, Shi et al. (2007) reported a decrease in psbA and psbE (PSII), psaA and psaC (PSI), petB and petC (Cyt *b*_6_*f* complex) and *nifH* (nitrogenase); where expression of *nifH* decreased significantly more than PSI or PSII genes. By contrast, Küpper et al. (2008) found that both the abundance of iron requiring PSI and the total phycobiliproteins measured from single-cell *in vivo* spectra remained constant under iron-limitation. The selective decrease in *nifH* over genes encoding for components of the photosynthetic machinery of the electron transport chain may aid in reducing the risk of photodamage and conserve energy which can be used to increase the carbon concentrating mechanism (CCM), CO_2_ fixation or up-regulate photoprotective (i.e., IdiA and IsiA) or iron-scavenging (i.e., TonB, ExbB, and ExbD) proteins.

Proteins associated with N_2_ fixation are more affected under iron-limitation than those associated with photosynthesis (Paerl et al., 1994; Shi et al., 2007; Brown et al., 2008; Fu et al., 2008; Küpper et al., 2008). Decreased nitrogenase activity induced by iron-limitation (Berman-Frank et al., 2001, 2007) decreases a major sink for reductant and energy that is otherwise supplied by respiratory electron flow through the Cyt *b6f* complex. This complex couples PSII to PSI by transferring electrons from hydroplastoquinone (PQH_2_) to plastocyanin (PC) (Supplementary Figure 3). Thus, under iron-limitation, electrons originating from photosynthesis (oxidation of water) and respiration (oxidation of organic carbon) could be bottlenecked at the Cyt *b6f* complex, resulting in a highly reduced plastoquinone (PQ) pool and consequent decrease in non-*F*_*b*_ corrected *F*_*v*_/*F*_*m*_ (Supplementary Figure 1).

Alternatively, under iron-limited conditions, it may be that photoinactivated PSII reaction centres accumulate within the thylakoid membrane, which could account for the lower values for *F*_*v*_/*F*_*m*_. In addition, connectivity between active and photoinactivated PSIIs within a dimer or less efficient connectivity among monomeric PSIIs could account for the increase in σ_PII_ observed at very low iron concentrations (Figure 1).

Additionally, non-*F*_*b*_ corrected *F*_*v*_/*F*_*m*_ may also decrease in part to an increased expression of IsiA, which forms an antenna around PSI, increasing the absorption cross-sections of PSI light harvesting (σ_LHI_) (Bibby et al., 2001a,b; Melkozernov and Blankenship, 2005; Wang et al., 2008). This mechanism may also play a critical role in non-photochemical quenching as (i), blue light converts IsiA from one form (efficient in harvesting photons) to another (converts excess energy to heat) (Cadoret et al., 2004) and (ii), high light increases the affinity of IsiA for phycobilisomes thus reducing the high fluorescence of free phycobilisomes (Joshua et al., 2005). In addition, IsiA can aggregate to form empty multimeric rings (without PSI) which exhibits a strong quenched state (Yeremenko et al., 2004), and are responsible for the dissipation of thermal energy (Ihalainen et al., 2005).

### Iron, CO_2_, and light dependencies on photophysiology

Under iron-limitation, the initial slopes of the rP-light curves declined due to a decrease in *F*_*q*_′/*F*_*m*_′ (Supplementary Figure 4). Due to light-dependent state transitions, *F*_*o*_′ can't be calculated using the initial, pre-FLC dark step measure of *F*_*o*_ and *F*_*v*_*/F*_*m*_ only; and as such *F*_*q*_′/*F*_*m*_′ was not separated into its two contributing processes; the PSII photochemical efficiency factor (*F*_*q*_′/*F*_*v*_′) and the maximum efficiency of PSII photochemistry (*F*_*v*_′/*F*_*m*_′). The lower operating efficiency of PSII (*F*_*q*′_/*F*_*m*_′), and subsequent lower rP at low CO_2_ is likely attributed to the an up-regulated CCM and/or to a down-regulation of the IdiA protein, which is thought to maintain optimal PSII activity (Michel and Pistorius, 2004).

*Trichodesmium* has high PSI:PSII ratios ranging between 1.3 and 4 under iron-replete diazotrophic conditions (Berman-Frank et al., 2001, 2007; Levitan et al., 2007, 2010; Brown et al., 2008). Previous studies have shown PSII to be less sensitive to iron-limitation than PSI at both an mRNA and protein level (Richier et al., 2012). It has been proposed that decreasing the PSI:PSII ratio is a physiological response to conserve iron (Berman-Frank et al., 2001), and one which could be compensated by increasing the cross-section of PSI light harvesting via IsiA-PSI super-complexes (Bibby et al., 2001a; Ryan-Keogh et al., 2012).

Pseudocyclic electron transport describes the movement of electrons around PSI, and the Mehler reaction (via flavodoxin) which is a photo-catalysed reaction consuming O_2_ (helping prevent nitrogenase inhibition) whilst simultaneously supplying ATP to nitrogenase (Supplementary Figure 3). Mehler activity can consume up to 75% of the O_2_ evolved from PSII (Milligan et al., 2007). Prolonged exposure to iron-limiting concentrations could lead to the occurrence of reactive oxygen species as several key proteins (e.g., catalase, peroxidise, superoxidase dismutase) associated to the Mehler reaction are dependent on iron as a co-factor. However, as the nitrogenase pool is significantly reduced under iron-limitation, maintaining the same degree of intracellular anoxia may be less critical.

As reported here, the PSII operating efficiency decreased significantly under iron-limitation, yielding lower rP_m_. Given that IsiA proteins can be 4 and 6 times more abundant than PSI and PSII proteins in iron-starved cultures and natural populations, respectively (Richier et al., 2012), we suggest that under iron-limitation, *Trichodesmium* increases pseudocyclic electron transport, with energy being re-directed from nitrogenase to enhance production of key proteins associated with iron stress (i.e., IsiA and IsiD).

## Conclusion

Our findings highlight iron as a major influencing factor on *Trichodesmium* growth, productivity and biogeographical distribution. Whilst iron-limitation constrains maximal *Trichodesmium* growth and productivity in the open ocean (Rueter, 1988; Rueter et al., 1990), integrated effects of elevated CO_2_ and/or high light intensities may act as negative feedbacks to climate change. Under iron-limitation the positive effects of elevated CO_2_ likely arise from a down-regulation of the CCM, whilst the positive effects of saturating light are likely due to a decrease in the requirement for key metalloenzymes, and lead to an increase in iron scavenging mechanisms and high light induced proteins (HLIP). Our findings are important given predictions of an increase in water stratification, a decrease in upwelling and wind-driven mixing, a shoaling of the mixed layer depth, increases in CO_2_ and sea surface temperatures (SSTs), as well as higher daily light exposures within the water column over the coming decades (Doney, 2006).

A major source of both iron and phosphorus to the Atlantic Ocean is Aeolian dust, which provides up to 16 Tg Fe yr^−1^ (Jickells et al., 2005) and 1.15 Tg P yr^−1^ (Mahowald et al., 2008), or 82% and 83% of total input, respectively. Although toxic to picoeukaryotes and *Synechococcus* (Paytan et al., 2009), Aeolian dust benefits *Trichodesmium* by providing a source of bioavailable iron (Carpenter and Romans, 1991) coupled with a low N:P ratio. This combination of features stimulates diazotrophic growth without providing a competitive advantage to other phytoplankton groups (Krishnamurthy et al., 2010).

One might expect changes in light and temperature to have a minimal direct effect on iron concentrations in the ocean. However, increased light intensity will increase Fe′ through the photolysis of ferric chelates and the resulting iron redox cycle. Temperature also influences the light requirement for algal growth, as light absorption by photosynthetic pigments and associated photochemistry within the photosynthetic reaction centres are insensitive to temperature, whereas downstream “dark” metabolic reactions which support growth are highly temperature dependent (Geider, 1987; Raven and Geider, 1988). Consequently, higher light is needed to support growth at a higher temperature (Davison, 1991). As such, increased CO_2_ and higher light intensities in the surface waters could help enhance *Trichodesmium's* productivity and growth in the future, potentially expanding its distribution into more iron-limited regions. However, it is worth considering whether the direct and indirect benefits of elevated CO_2_ and higher light intensity outweigh the disadvantages of supra-optimal SSTs.

## Author contributions

TB and RG: Conceptualisation. TB, MG, and RG: Methodology. TB and MG: Software. TB and RG: Validation. TB and KO: Formal analysis. TB: Investigation. TB and RG: Resources. TB: Data curation. TB: Writing (original draft preparation). TB, KO, MG, and RG: Writing (review and editing). TB: Visualisation. RG and TL: Supervision. TB: Project administration. RG and TL: Funding acquisition.

### Conflict of interest statement

The authors declare that the research was conducted in the absence of any commercial or financial relationships that could be construed as a potential conflict of interest.
