# Supplementary material for: An Integrated Response of Trichodesmium erythraeum IMS101 Growth and Photo-Physiology to Iron, CO2, and Light Intensity
Source: Front Microbiol. 2018 Apr 10;9:624. doi: 10.3389/fmicb.2018.00624 (PMC5932364; doi:10.3389/fmicb.2018.00624)
Supplement: Supplementary file 1 [file Table1.pdf]

Supplementary Table 1. The total (Fe_T) and free (Fe′) iron concentrations (±S.E.) of the various CO_2 and light treatments.

		Low CO_2		Mid CO_2		High CO_2	
**Parameters**	**Fe_T**	**LL**	**HL**	**LL**	**HL**	**LL**	**HL**
Light response	400	5507 (952)	5559 (319)	6440 (391)	3694 (445)	3887 (256)	9676 (421)
Temperature response	400	4938 (136)	5601 (1)	4513 (385)	6200 (448)	3950 (248)	8909 (135)
Iron response	400	288 (24)	550 (16)	393 (14)	562 (19)	355 (13)	853 (5)
	200	444 (24)	489 (22)	347 (21)	649 (34)	350 (26)	1037 (3)
	100	435 (60)	563 (45)	335 (8)	596 (22)	205 (4)	478 (12)
	40	494 (12)	539 (26)	357 (6)	597 (21)	102 (4)	289 (9)
	4		239 (16)		66 (1)		23 (1)

Light and temperature response cultures used a 2 µM EDTA concentration, as reported in Boatman et al. (2017). The iron response cultures used a 20 µM EDTA concentration.
