# Supplementary material for: An Integrated Response of Trichodesmium erythraeum IMS101 Growth and Photo-Physiology to Iron, CO2, and Light Intensity
Source: Front Microbiol. 2018 Apr 10;9:624. doi: 10.3389/fmicb.2018.00624 (PMC5932364; doi:10.3389/fmicb.2018.00624)
Supplement: Supplementary file 2 [file Table2.pdf]

Supplementary Table 2. The variance of separated-CO_2 (e.g. Mid) and combined-CO_2 (e.g. Low + Mid) growth rate-Fe′ curves; where μ_m and K_m were modelled using a Michaelis-Menten equation (Michaelis & Menten, 1913).

	μ_m	S.E.	*t*-value	*p*	K_m	S.E.	*t*-value	*p*	RSS	df
Low Light										
LCO_2	0.104	0.0053	19.56	< 0.0001	306.98	56.21	5.46	0.0055	0.0002	4
MCO_2	0.136	0.0062	21.85	< 0.0001	116.36	29.31	3.97	0.0165	0.0002	4
HCO_2	0.134	0.0120	11.23	0.0004	123.97	47.51	2.61	0.0594	0.0010	4
LCO_2 + MCO_2	0.115	0.0135	8.55	< 0.0001	156.19	87.14	1.79	0.1033	0.0056	10
LCO_2 + HCO_2	0.112	0.0133	8.44	< 0.0001	144.67	78.38	1.85	0.0947	0.0058	10
MCO_2 + HCO_2	0.134	0.0006	21.70	< 0.0001	117.49	26.45	4.44	0.0013	0.0013	10
LCO_2 + MCO_2 + HCO_2	0.119	0.0100	11.90	< 0.0001	131.00	53.89	2.43	0.0272	0.0080	16
High Light										
LCO_2	0.193	0.0211	9.16	0.0003	236.14	104.16	2.27	0.0727	0.0033	5
MCO_2	0.337	0.0245	13.74	< 0.0001	205.80	71.16	2.89	0.0341	0.0045	5
HCO_2	0.367	0.0161	22.71	< 0.0001	201.19	45.86	4.37	0.0071	0.0025	5
LCO_2 + MCO_2	0.270	0.0395	6.83	< 0.0001	241.81	148.01	1.63	0.1283	0.0538	12
LCO_2 + HCO_2	0.295	0.0434	6.78	< 0.0001	290.68	171.03	1.70	0.1150	0.0712	12
MCO_2 + HCO_2	0.354	0.0148	23.84	0.0001	209.68	42.96	4.88	0.0004	0.0090	12
LCO_2 + MCO_2 + HCO_2	0.309	0.0314	9.82	< 0.0001	256.72	110.58	2.32	0.0315	0.0865	19

An *F*-statistic was calculated as the (RSS/df) of a separate fit, divided by the (RSS/df) of the difference between the separate and combined fit; this was then compared against an *F*-value from an *F*-distribution table using a .05 alpha level.
