# Supplementary material for: An Integrated Response of Trichodesmium erythraeum IMS101 Growth and Photo-Physiology to Iron, CO2, and Light Intensity
Source: Front Microbiol. 2018 Apr 10;9:624. doi: 10.3389/fmicb.2018.00624 (PMC5932364; doi:10.3389/fmicb.2018.00624)
Supplement: Supplementary file 3 [file Table3.pdf]

Supplementary Table 3. The variance of separated-CO_2 (e.g. Mid) and combined-CO_2 (e.g. Low + Mid) growth rate-Fe′ curves, modelled using a Michaelis-Menten equation (Michaelis & Menten, 1913); where each curve was normalised to the modelled maximum growth rate (μ_m) for that CO_2 and light treatment.

	**μ_m**	**S.E.**	***t*-value**	***p***	**K_m**	**S.E.**	***t*-value**	***p***	**RSS**	**df**
Low Light										
LCO_2	1.0	0.0464	21.55	< 0.0001	309.95	51.17	6.06	0.0038	0.0114	4
MCO_2	1.0	0.0523	19.13	< 0.0001	104.16	32.26	3.23	0.0320	0.0176	4
HCO_2	1.0	0.0876	11.41	0.0003	125.21	47.28	2.65	0.0571	0.0519	4
LCO_2 + MCO_2	1.0	0.0676	14.78	< 0.0001	182.71	54.06	3.38	0.0070	0.1376	10
LCO_2 + HCO_2	1.0	0.0712	14.05	< 0.0001	188.75	54.84	3.44	0.0063	0.1537	10
MCO_2 + HCO_2	1.0	0.0459	21.80	< 0.0001	115.78	26.21	4.42	0.0013	0.0720	10
LCO_2 + MCO_2 + HCO_2	1.0	0.0525	19.03	< 0.0001	157.06	36.90	4.26	0.0006	0.2098	16
High Light										
LCO_2	1.0	0.1104	9.06	0.0003	232.91	103.87	2.24	0.0750	0.0907	5
MCO_2	1.0	0.0765	13.07	< 0.0001	206.41	75.27	2.74	0.0407	0.0441	5
HCO_2	1.0	0.0445	22.46	< 0.0001	201.24	46.24	4.34	0.0075	0.0192	5
LCO_2 + MCO_2	1.0	0.0617	16.21	< 0.0001	213.24	58.36	3.65	0.0033	0.1378	12
LCO_2 + HCO_2	1.0	0.0523	19.11	< 0.0001	219.54	51.79	4.24	0.0011	0.1119	12
MCO_2 + HCO_2	1.0	0.0385	25.97	< 0.0001	195.68	38.11	5.13	0.0002	0.0625	12
LCO_2 + MCO_2 + HCO_2	1.0	0.0409	24.44	< 0.0001	210.06	39.92	5.26	< 0.0001	0.1574	19

An *F*-statistic was calculated as the (RSS/df) of a separate fit, divided by the (RSS/df) of the difference between the separate and combined fit; this was then compared against an *F*-value from an *F*-distribution table using a .05 alpha level.
