# Supplementary material for: An Integrated Response of Trichodesmium erythraeum IMS101 Growth and Photo-Physiology to Iron, CO2, and Light Intensity
Source: Front Microbiol. 2018 Apr 10;9:624. doi: 10.3389/fmicb.2018.00624 (PMC5932364; doi:10.3389/fmicb.2018.00624)
Supplement: Supplementary file 4 [file Table4.pdf]

Supplementary Table 4. The variance of separated-CO_2 (e.g. Mid) and combined-CO_2 (e.g. Low + Mid) growth rate-Fe′ curves, modelled using a Michaelis-Menten equation (Michaelis & Menten, 1913); where each curve was normalised to the modelled half saturation parameter (K_m) for that CO_2 and light treatment.

	μ_m	S.E.	*t*-value	*p*	K_m	S.E.	*t*-value	*p*	RSS	df
Low Light										
LCO_2	0.104	0.0048	21.66	< 0.0001	1.0	0.1642	6.09	0.0037	0.0001	4
MCO_2	0.132	0.0069	19.12	< 0.0001	1.0	0.3101	3.23	0.0321	0.0003	4
HCO_2	0.134	0.0117	11.45	0.0003	1.0	0.3766	2.66	0.0567	0.0009	4
LCO_2 + MCO_2	0.121	0.0074	16.23	< 0.0001	1.0	0.2770	3.61	0.0048	0.0018	10
LCO_2 + HCO_2	0.121	0.0091	13.17	< 0.0001	1.0	0.2967	3.37	0.0071	0.0025	10
MCO_2 + HCO_2	0.133	0.0060	22.23	< 0.0001	1.0	0.2253	4.44	0.0013	0.0012	10
LCO_2 + MCO_2 + HCO_2	0.125	0.0063	19.71	< 0.0001	1.0	0.2249	4.45	0.0004	0.0032	16
High Light										
LCO_2	0.193	0.0213	9.077	0.0003	1.0	0.4459	2.24	0.0750	0.0034	5
MCO_2	0.337	0.0247	13.647	< 0.0001	1.0	0.3496	2.86	0.0354	0.0046	5
HCO_2	0.367	0.0163	22.456	< 0.0001	1.0	0.2308	4.34	0.0075	0.0026	5
LCO_2 + MCO_2	0.266	0.0371	7.172	< 0.0001	1.0	0.6092	1.64	0.1266	0.0500	12
LCO_2 + HCO_2	0.281	0.0403	6.973	< 0.0001	1.0	0.6478	1.54	0.1486	0.0674	12
MCO_2 + HCO_2	0.352	0.0148	23.813	< 0.0001	1.0	0.2084	4.80	0.0004	0.0090	12
LCO_2 + MCO_2 + HCO_2	0.300	0.0292	10.280	< 0.0001	1.0	0.4473	2.24	0.0376	0.0806	19

An *F*-statistic was calculated as the (RSS/df) of a separate fit, divided by the (RSS/df) of the difference between the separate and combined fit; this was then compared against an *F*-value from an *F*-distribution table using a .05 alpha level.
