# Supplementary material for: An Integrated Response of Trichodesmium erythraeum IMS101 Growth and Photo-Physiology to Iron, CO2, and Light Intensity
Source: Front Microbiol. 2018 Apr 10;9:624. doi: 10.3389/fmicb.2018.00624 (PMC5932364; doi:10.3389/fmicb.2018.00624)
Supplement: Supplementary file 5 [file Table5.pdf]

Supplementary Table 5. The variance of separated-CO_2 (e.g. Mid) and combined-CO_2 (e.g. Low + Mid) growth rate-Fe′ curves, modelled using a P-E equation (Michaelis & Menten, 1913; Platt & Gallegos, 1980); where each curve was normalised to the modelled maximum growth rate (μ_m) for that CO_2 and light treatment.

	μ_m	S.E.	*t*-value	*p*	α	S.E.	*t*-value	*p*	RSS	df
Low Light										
LCO_2	1.0	0.0049	20.33	< 0.0001	0.0022	0.0002	9.85	0.0006	0.0195	4
MCO_2	1.0	0.0042	24.02	< 0.0001	0.0044	0.0004	9.74	0.0006	0.0139	4
HCO_2	1.0	0.0050	20.13	< 0.0001	0.0051	0.0007	7.80	0.0015	0.0214	4
LCO_2 + MCO_2	1.0	0.0056	17.66	< 0.0001	0.0031	0.0004	8.04	< 0.0001	0.1271	10
LCO_2 + HCO_2	1.0	0.0069	14.04	< 0.0001	0.0035	0.0006	5.83	0.0002	0.1968	10
MCO_2 + HCO_2	1.0	0.0033	30.52	< 0.0001	0.0046	0.0004	11.64	< 0.0001	0.0444	10
LCO_2 + MCO_2 + HCO_2	1.0	0.0048	20.51	< 0.0001	0.0037	0.0004	8.44	< 0.0001	0.2245	16
High Light										
LCO_2	1.0	0.0783	12.77	< 0.0001	0.0027	0.0005	5.87	0.0020	0.0634	5
MCO_2	1.0	0.0642	15.57	< 0.0001	0.0028	0.0005	5.82	0.0021	0.0423	5
HCO_2	1.0	0.0402	24.93	< 0.0001	0.0028	0.0004	7.92	0.0005	0.0206	5
LCO_2 + MCO_2	1.0	0.0460	21.73	< 0.0001	0.0027	0.0003	9.07	< 0.0001	0.1057	12
LCO_2 + HCO_2	1.0	0.0389	25.73	< 0.0001	0.0028	0.0003	10.04	< 0.0001	0.0842	12
MCO_2 + HCO_2	1.0	0.0338	29.58	< 0.0001	0.0028	0.0003	10.23	< 0.0001	0.0631	12
LCO_2 + MCO_2 + HCO_2	1.0	0.0315	31.76	< 0.0001	0.0028	0.0002	12.22	< 0.0001	0.1265	19

An *F*-statistic was calculated as the (RSS/df) of a separate fit, divided by the (RSS/df) of the difference between the separate and combined fit; this was then compared against an *F*-value from an *F*-distribution table using a .05 alpha level.
