# Supplementary material for: An Integrated Response of Trichodesmium erythraeum IMS101 Growth and Photo-Physiology to Iron, CO2, and Light Intensity
Source: Front Microbiol. 2018 Apr 10;9:624. doi: 10.3389/fmicb.2018.00624 (PMC5932364; doi:10.3389/fmicb.2018.00624)
Supplement: Supplementary file 6 [file Table6.pdf]

Supplementary Table 6. The variance of separated-CO_2 (e.g. Mid) and combined-CO_2 (e.g. Low + Mid) maximum relative PSII electron transport (rP_m)-Fe′ curves; where rP_m and K_m^{rPm} were modelled using a Michaelis-Menten equation (Michaelis & Menten, 1913).

	rP_m	S.E.	*t*-value	*p*	K_m^{rPm}	S.E.	*t*-value	*p*	RSS	df
Low Light										
LCO_2	152.37	11.20	13.61	0.0009	21.88	35.01	0.62	0.5763	361.4	3
MCO_2	153.72	26.42	5.82	0.0101	21.73	74.75	0.29	0.7902	1860.9	3
HCO_2	162.00	14.64	11.07	0.0016	35.65	24.92	1.43	0.2480	916.4	3
LCO_2 + MCO_2	152.91	12.22	12.51	< 0.0001	21.36	36.27	0.59	0.5721	2226.7	8
LCO_2 + HCO_2	157.26	7.35	21.38	< 0.0001	31.03	15.64	1.98	0.0825	1348.3	8
MCO_2 + HCO_2	158.47	11.03	14.37	< 0.0001	31.85	22.83	140	0.2004	2821.0	8
LCO_2 + MCO_2 + HCO_2	156.59	7.54	20.76	< 0.0001	29.73	17.33	1.72	0.1100	3219.1	13
High Light										
LCO_2	233.41	27.04	8.63	0.0010	151.39	79.82	1.90	0.1307	2679.0	4
MCO_2	222.82	6.26	35.58	<0 .0001	77.08	14.24	5.41	0.0056	317.3	4
HCO_2	211.81	19.42	10.90	0.0004	17.16	13.92	1.23	0.2851	6101.8	4
LCO_2 + MCO_2	225.97	12.27	18.42	< 0.0001	107.84	33.60	3.21	0.0093	3836.2	10
LCO_2 + HCO_2	202.29	13.71	14.75	< 0.0001	23.34	17.56	1.33	0.2133	13597.5	10
MCO_2 + HCO_2	214.92	12.12	17.74	< 0.0001	38.56	18.03	2.14	0.0582	9070.5	10
LCO_2 + MCO_2 + HCO_2	217.56	12.27	17.73	< 0.0001	64.32	27.41	2.35	0.0322	14647.6	16

An *F*-statistic was calculated as the (RSS/df) of a separate fit, divided by the (RSS/df) of the difference between the separate and combined fit; this was then compared against an *F*-value from an *F*-distribution table using a .05 alpha level.
