# Supplementary material for: An Integrated Response of Trichodesmium erythraeum IMS101 Growth and Photo-Physiology to Iron, CO2, and Light Intensity
Source: Front Microbiol. 2018 Apr 10;9:624. doi: 10.3389/fmicb.2018.00624 (PMC5932364; doi:10.3389/fmicb.2018.00624)
Supplement: Supplementary file 7 [file Table7.pdf]

Supplementary Table 7. The variance of separated-CO_2 (e.g. Mid) and combined-CO_2 (e.g. Low + Mid) maximum relative PSII electron transport-Fe′ curves, modelled using a Michaelis-Menten equation (Michaelis & Menten, 1913); where each curve was normalised to the modelled maximum relative PSII electron transport (rP_m) for that CO_2 and light treatment.

	rP_m	S.E.	*t*-value	*p*	K_m^{rPm}	S.E.	*t*-value	*p*	RSS	df
Low Light										
LCO_2	1.0	0.07	13.61	0.0009	21.88	35.01	0.62	0.5763	0.0156	3
MCO_2	1.0	0.17	5.82	0.0101	21.73	74.75	0.29	0.7902	0.0788	3
HCO_2	1.0	0.09	11.07	0.0016	35.65	24.92	1.43	0.2480	0.0349	3
LCO_2 + MCO_2	1.0	0.08	12.57	< 0.0001	21.78	36.15	0.60	0.5635	0.0943	8
LCO_2 + HCO_2	1.0	0.05	21.65	< 0.0001	35.80	16.00	2.24	0.0556	0.0526	8
MCO_2 + HCO_2	1.0	0.07	14.13	< 0.0001	35.22	23.78	1.48	0.1769	0.1163	8
LCO_2 + MCO_2 + HCO_2	1.0	0.05	20.56	< 0.0001	34.29	18.03	1.90	0.0795	0.1329	13
High Light										
LCO_2	1.0	0.16	8.63	0.0010	151.39	79.82	1.90	0.1307	0.0492	4
MCO_2	1.0	0.03	35.58	< 0.0001	77.08	14.24	5.41	0.0056	0.0064	4
HCO_2	1.0	0.09	10.90	0.0004	17.92	13.92	1.23	0.2851	0.1360	4
LCO_2 + MCO_2	1.0	0.06	17.02	< 0.0001	109.05	36.53	2.99	0.0137	0.0859	10
LCO_2 + HCO_2	1.0	0.07	13.46	< 0.0001	19.48	16.87	1.16	0.2749	0.3477	10
MCO_2 + HCO_2	1.0	0.06	16.67	< 0.0001	37.84	18.93	2.00	0.0735	0.2191	10
LCO_2 + MCO_2 + HCO_2	1.0	0.06	15.77	< 0.0001	64.47	30.87	2.09	0.0531	0.3743	16

An *F*-statistic was calculated as the (RSS/df) of a separate fit, divided by the (RSS/df) of the difference between the separate and combined fit; this was then compared against an *F*-value from an *F*-distribution table using a .05 alpha level.
