# Supplementary material for: An Integrated Response of Trichodesmium erythraeum IMS101 Growth and Photo-Physiology to Iron, CO2, and Light Intensity
Source: Front Microbiol. 2018 Apr 10;9:624. doi: 10.3389/fmicb.2018.00624 (PMC5932364; doi:10.3389/fmicb.2018.00624)
Supplement: Supplementary file 8 [file Table8.pdf]

Supplementary Table 8. Mean dissolved inorganic N concentrations (µM) measured from Fe-replete (2 µM EDTA), optimal growth temperature cultures reported in Boatman et al. (2017).

		**Low CO_2**		**Mid CO_2**		**High CO_2**	
**Parameters**	**Units**	**LL**	**HL**	**LL**	**HL**	**LL**	**HL**
Temperature response							
NH_4^+	µM	1.66 (0.52)	1.00 (0.31)	0.92 (0.20)	0.90 (0.20)	1.04 (0.22)	1.13 (0.24)
NO_3^-	µM	0.11 (0.01)	0.21 (0.06)	0.19 (0.04)	0.21 (0.05)	0.14 (0.03)	0.20 (0.04)
Light response							
NH_4^+	µM	0.90 (0.07)	1.04 (0.30)	0.69 (0.21)	0.94 (0.28)	0.85 (0.25)	0.94 (0.11)
NO_3^-	µM	0.09 (0.01)	0.15 (0.04)	0.09 (0.03)	0.17 (0.05)	0.13 (0.04)	0.18 (0.02)

T. erythraeum IMS101 cultures were fully acclimated to three CO_2 concentrations (Low CO_2 = 180 µatm, Mid CO_2 = 380 µatm and High CO_2 = 720 µatm), two light intensities (LL = 40 µmol photons m^{-2} s^{-1}; HL = 400 µmol photons m^{-2} s^{-1}), under Fe replete conditions at optimal temperature (26.2 °C). Nitrate concentrations were measured spectrophotometrically as described by Collos *et al.* (1999), and were always less than the detection limit (1 µM) of the method.
