# Supplementary material for: An Integrated Response of Trichodesmium erythraeum IMS101 Growth and Photo-Physiology to Iron, CO2, and Light Intensity
Source: Front Microbiol. 2018 Apr 10;9:624. doi: 10.3389/fmicb.2018.00624 (PMC5932364; doi:10.3389/fmicb.2018.00624)
Supplement: Supplementary file 9 [file Image1.pdf]

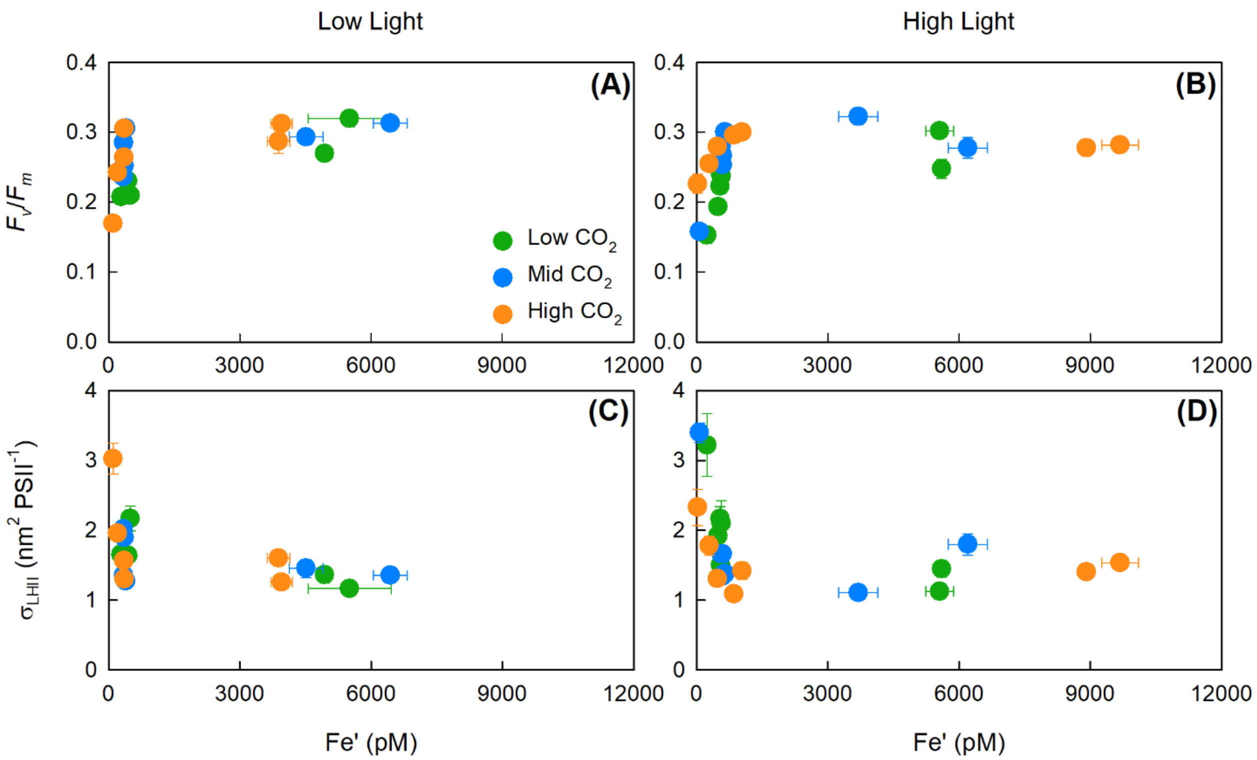

Supplementary Figure 1. The mean (± S.E.) non-baseline (F_b) corrected photochemical efficiency of PSII in the dark-acclimated state (F_v/F_m) (A,B) and absorption cross section of PSII photochemistry ($\sigma_{LHII} = \sigma_{PII}$ / F_v/F_m) for *T. erythraeum* IMS101. Left-hand panels (A,C) are low light treatments while right-hand panels (B,D) are high light treatments. Cultures were acclimated to three targeted CO_2 concentrations (Low = 180 µatm (green circles), Mid = 380 µatm (blue circles) and High = 720 µatm (orange circles)), two light intensities (LL = 40 µmol photons m^{-2} s^{-1}, HL = 400 µmol photons m^{-2} s^{-1}), across a range of Fe′ concentrations (~ 20 - 9600 pM) and at optimal temperature (26 °C).
