# Supplementary figures and images for: An Integrated Response of Trichodesmium erythraeum IMS101 Growth and Photo-Physiology to Iron, CO2, and Light Intensity
Source: Front Microbiol. 2018 Apr 10;9:624. doi: 10.3389/fmicb.2018.00624 (PMC5932364; doi:10.3389/fmicb.2018.00624)

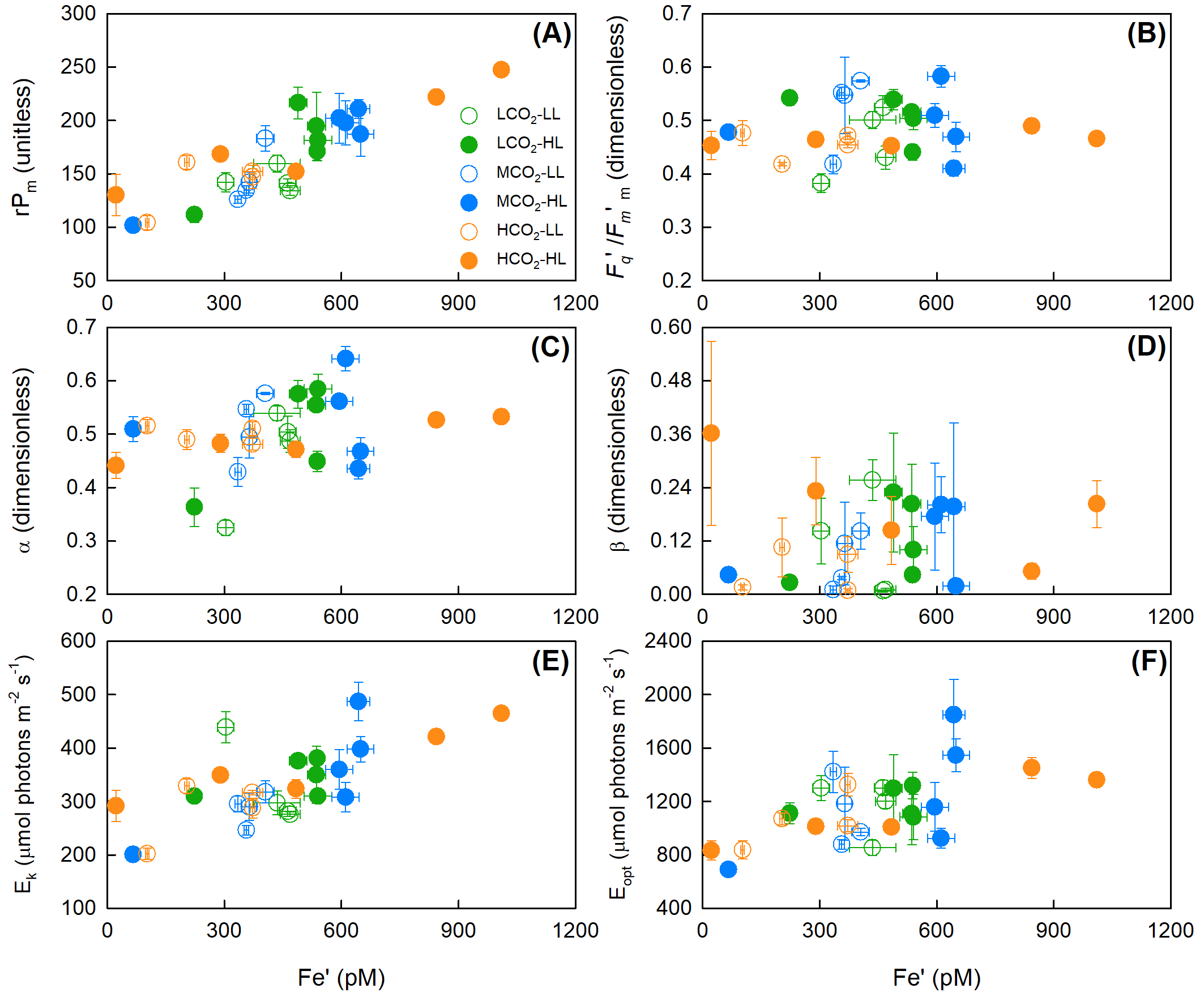

Supplement: Supplementary file 10 [file Image2.TIF]

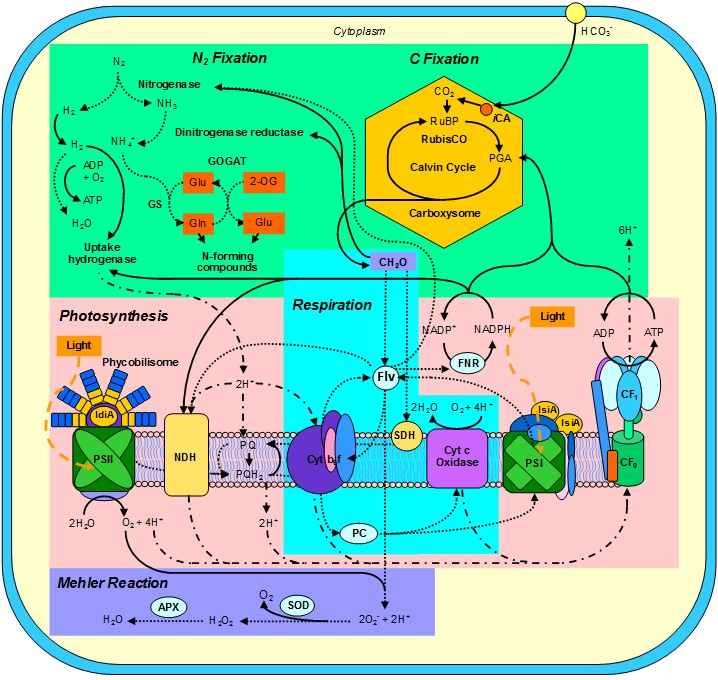

Supplement: Supplementary file 11 [file Image3.JPEG]

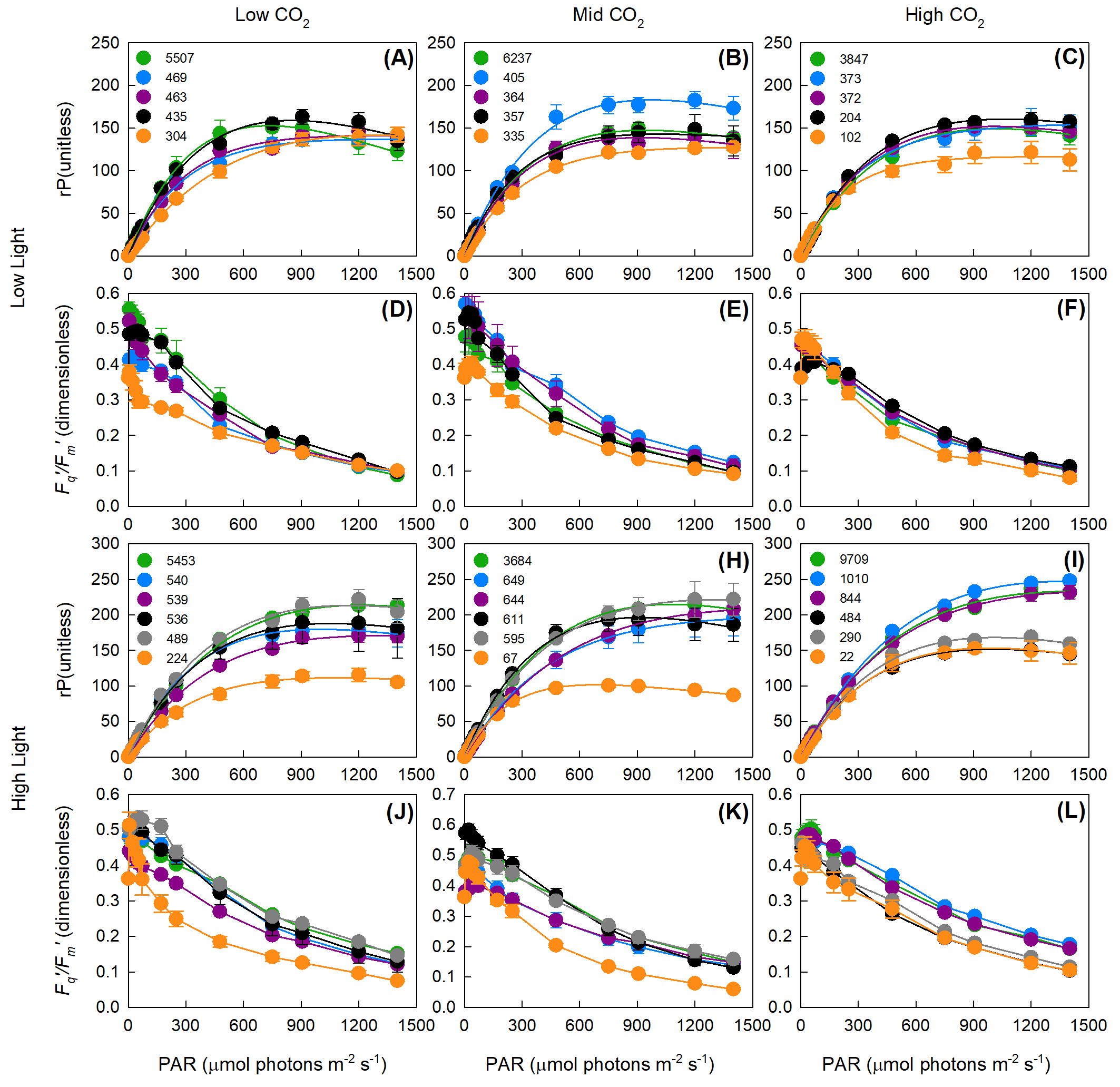

Supplement: Supplementary file 12 [file Image4.TIF]
