# Supplementary material for: An Integrated Response of Trichodesmium erythraeum IMS101 Growth and Photo-Physiology to Iron, CO2, and Light Intensity
Source: Front Microbiol. 2018 Apr 10;9:624. doi: 10.3389/fmicb.2018.00624 (PMC5932364; doi:10.3389/fmicb.2018.00624)
Supplement: Supplementary file 13 [file Table1.docx]

Supplementary Information

An integrated response of *Trichodesmium erythraeum* IMS101 growth and photo-physiology to iron, CO_2_ and light intensity

Tobias G. Boatman^*^, Kevin Oxborough, Martha Gledhill, Tracy Lawson, Richard J. Geider

*** Correspondence:** Tobias Boatman [tboatman@chelsea.co.uk](mailto:tboatman@chelsea.co.uk)

**Supplementary Table 1. The total (Fe_T_) and free (Feʹ) iron concentrations (±S.E.) of the various CO_2_ and light treatments.**

|  |  | **Low CO_2_** | | **Mid CO_2_** | | **High CO_2_** | |
| --- | --- | --- | --- | --- | --- | --- | --- |
| **Parameters** | **Fe_T_** | **LL** | **HL** | **LL** | **HL** | **LL** | **HL** |
| Light response | 400 | 5507 (952) | 5559 (319) | 6440 (391) | 3694 (445) | 3887 (256) | 9676 (421) |
| Temperature response | 400 | 4938 (136) | 5601 (1) | 4513 (385) | 6200 (448) | 3950 (248) | 8909 (135) |
| Iron response | 400 | 288 (24) | 550 (16) | 393 (14) | 562 (19) | 355 (13) | 853 (5) |
|  | 200 | 444 (24) | 489 (22) | 347 (21) | 649 (34) | 350 (26) | 1037 (3) |
|  | 100 | 435 (60) | 563 (45) | 335 (8) | 596 (22) | 205 (4) | 478 (12) |
|  | 40 | 494 (12) | 539 (26) | 357 (6) | 597 (21) | 102 (4) | 289 (9) |
|  | 4 |  | 239 (16) |  | 66 (1) |  | 23 (1) |

Light and temperature response cultures used a 2 µM EDTA concentration, as reported in [Boatman et al. (2017](#_ENREF_2)). The iron response cultures used a 20 µM EDTA concentration.

**Supplementary Table 2. The variance of separated-CO_2_ (e.g. Mid) and combined-CO_2_ (e.g. Low + Mid) growth rate-Feʹ curves; where µ_m_ and K_m_ were modelled using a Michaelis-Menten equation (**[**Michaelis & Menten, 1913**](#_ENREF_5)**).**

|  | **µ_m_** | **S.E.** | ***t*-value** | ***p*** | **K_m_** | **S.E.** | ***t*-value** | ***p*** | **RSS** | **df** |
| --- | --- | --- | --- | --- | --- | --- | --- | --- | --- | --- |
| Low Light |  |  |  |  |  |  |  |  |  |  |
| LCO_2_ | 0.104 | 0.0053 | 19.56 | < 0.0001 | 306.98 | 56.21 | 5.46 | 0.0055 | 0.0002 | 4 |
| MCO_2_ | 0.136 | 0.0062 | 21.85 | < 0.0001 | 116.36 | 29.31 | 3.97 | 0.0165 | 0.0002 | 4 |
| HCO_2_ | 0.134 | 0.0120 | 11.23 | 0.0004 | 123.97 | 47.51 | 2.61 | 0.0594 | 0.0010 | 4 |
| LCO_2_ + MCO_2_ | 0.115 | 0.0135 | 8.55 | < 0.0001 | 156.19 | 87.14 | 1.79 | 0.1033 | 0.0056 | 10 |
| LCO_2_ + HCO_2_ | 0.112 | 0.0133 | 8.44 | < 0.0001 | 144.67 | 78.38 | 1.85 | 0.0947 | 0.0058 | 10 |
| MCO_2_ + HCO_2_ | 0.134 | 0.0006 | 21.70 | < 0.0001 | 117.49 | 26.45 | 4.44 | 0.0013 | 0.0013 | 10 |
| LCO_2_ + MCO_2_ + HCO_2_ | 0.119 | 0.0100 | 11.90 | < 0.0001 | 131.00 | 53.89 | 2.43 | 0.0272 | 0.0080 | 16 |
| High Light |  |  |  |  |  |  |  |  |  |  |
| LCO_2_ | 0.193 | 0.0211 | 9.16 | 0.0003 | 236.14 | 104.16 | 2.27 | 0.0727 | 0.0033 | 5 |
| MCO_2_ | 0.337 | 0.0245 | 13.74 | < 0.0001 | 205.80 | 71.16 | 2.89 | 0.0341 | 0.0045 | 5 |
| HCO_2_ | 0.367 | 0.0161 | 22.71 | < 0.0001 | 201.19 | 45.86 | 4.37 | 0.0071 | 0.0025 | 5 |
| LCO_2_ + MCO_2_ | 0.270 | 0.0395 | 6.83 | < 0.0001 | 241.81 | 148.01 | 1.63 | 0.1283 | 0.0538 | 12 |
| LCO_2_ + HCO_2_ | 0.295 | 0.0434 | 6.78 | < 0.0001 | 290.68 | 171.03 | 1.70 | 0.1150 | 0.0712 | 12 |
| MCO_2_ + HCO_2_ | 0.354 | 0.0148 | 23.84 | 0.0001 | 209.68 | 42.96 | 4.88 | 0.0004 | 0.0090 | 12 |
| LCO_2_ + MCO_2_ + HCO_2_ | 0.309 | 0.0314 | 9.82 | < 0.0001 | 256.72 | 110.58 | 2.32 | 0.0315 | 0.0865 | 19 |

An *F*-statistic was calculated as the (RSS/df) of a separate fit, divided by the (RSS/df) of the difference between the separate and combined fit; this was then compared against an *F*-value from an *F*-distribution table using a .05 alpha level.

**Supplementary Table 3. The variance of separated-CO_2_ (e.g. Mid) and combined-CO_2_ (e.g. Low + Mid) growth rate-Feʹ curves, modelled using a Michaelis-Menten equation (**[**Michaelis & Menten, 1913**](#_ENREF_5)**); where each curve was normalised to the modelled maximum growth rate (µ_m_) for that CO_2_ and light treatment.**

|  | **µ_m_** | **S.E.** | ***t*-value** | ***p*** | **K_m_** | **S.E.** | ***t*-value** | ***p*** | **RSS** | **df** |
| --- | --- | --- | --- | --- | --- | --- | --- | --- | --- | --- |
| Low Light |  |  |  |  |  |  |  |  |  |  |
| LCO_2_ | 1.0 | 0.0464 | 21.55 | < 0.0001 | 309.95 | 51.17 | 6.06 | 0.0038 | 0.0114 | 4 |
| MCO_2_ | 1.0 | 0.0523 | 19.13 | < 0.0001 | 104.16 | 32.26 | 3.23 | 0.0320 | 0.0176 | 4 |
| HCO_2_ | 1.0 | 0.0876 | 11.41 | 0.0003 | 125.21 | 47.28 | 2.65 | 0.0571 | 0.0519 | 4 |
| LCO_2_ + MCO_2_ | 1.0 | 0.0676 | 14.78 | < 0.0001 | 182.71 | 54.06 | 3.38 | 0.0070 | 0.1376 | 10 |
| LCO_2_ + HCO_2_ | 1.0 | 0.0712 | 14.05 | < 0.0001 | 188.75 | 54.84 | 3.44 | 0.0063 | 0.1537 | 10 |
| MCO_2_ + HCO_2_ | 1.0 | 0.0459 | 21.80 | < 0.0001 | 115.78 | 26.21 | 4.42 | 0.0013 | 0.0720 | 10 |
| LCO_2_ + MCO_2_ + HCO_2_ | 1.0 | 0.0525 | 19.03 | < 0.0001 | 157.06 | 36.90 | 4.26 | 0.0006 | 0.2098 | 16 |
| High Light |  |  |  |  |  |  |  |  |  |  |
| LCO_2_ | 1.0 | 0.1104 | 9.06 | 0.0003 | 232.91 | 103.87 | 2.24 | 0.0750 | 0.0907 | 5 |
| MCO_2_ | 1.0 | 0.0765 | 13.07 | < 0.0001 | 206.41 | 75.27 | 2.74 | 0.0407 | 0.0441 | 5 |
| HCO_2_ | 1.0 | 0.0445 | 22.46 | < 0.0001 | 201.24 | 46.24 | 4.34 | 0.0075 | 0.0192 | 5 |
| LCO_2_ + MCO_2_ | 1.0 | 0.0617 | 16.21 | < 0.0001 | 213.24 | 58.36 | 3.65 | 0.0033 | 0.1378 | 12 |
| LCO_2_ + HCO_2_ | 1.0 | 0.0523 | 19.11 | < 0.0001 | 219.54 | 51.79 | 4.24 | 0.0011 | 0.1119 | 12 |
| MCO_2_ + HCO_2_ | 1.0 | 0.0385 | 25.97 | < 0.0001 | 195.68 | 38.11 | 5.13 | 0.0002 | 0.0625 | 12 |
| LCO_2_ + MCO_2_ + HCO_2_ | 1.0 | 0.0409 | 24.44 | < 0.0001 | 210.06 | 39.92 | 5.26 | < 0.0001 | 0.1574 | 19 |

An *F*-statistic was calculated as the (RSS/df) of a separate fit, divided by the (RSS/df) of the difference between the separate and combined fit; this was then compared against an *F*-value from an *F*-distribution table using a .05 alpha level.

**Supplementary Table 4. The variance of separated-CO_2_ (e.g. Mid) and combined-CO_2_ (e.g. Low + Mid) growth rate-Feʹ curves, modelled using a Michaelis-Menten equation (**[**Michaelis & Menten, 1913**](#_ENREF_5)**); where each curve was normalised to the modelled half saturation parameter (K_m_) for that CO_2_ and light treatment.**

|  | **µ_m_** | **S.E.** | ***t*-value** | ***p*** | **K_m_** | **S.E.** | ***t*-value** | ***p*** | **RSS** | **df** |
| --- | --- | --- | --- | --- | --- | --- | --- | --- | --- | --- |
| Low Light |  |  |  |  |  |  |  |  |  |  |
| LCO_2_ | 0.104 | 0.0048 | 21.66 | < 0.0001 | 1.0 | 0.1642 | 6.09 | 0.0037 | 0.0001 | 4 |
| MCO_2_ | 0.132 | 0.0069 | 19.12 | < 0.0001 | 1.0 | 0.3101 | 3.23 | 0.0321 | 0.0003 | 4 |
| HCO_2_ | 0.134 | 0.0117 | 11.45 | 0.0003 | 1.0 | 0.3766 | 2.66 | 0.0567 | 0.0009 | 4 |
| LCO_2_ + MCO_2_ | 0.121 | 0.0074 | 16.23 | < 0.0001 | 1.0 | 0.2770 | 3.61 | 0.0048 | 0.0018 | 10 |
| LCO_2_ + HCO_2_ | 0.121 | 0.0091 | 13.17 | < 0.0001 | 1.0 | 0.2967 | 3.37 | 0.0071 | 0.0025 | 10 |
| MCO_2_ + HCO_2_ | 0.133 | 0.0060 | 22.23 | < 0.0001 | 1.0 | 0.2253 | 4.44 | 0.0013 | 0.0012 | 10 |
| LCO_2_ + MCO_2_ + HCO_2_ | 0.125 | 0.0063 | 19.71 | < 0.0001 | 1.0 | 0.2249 | 4.45 | 0.0004 | 0.0032 | 16 |
| High Light |  |  |  |  |  |  |  |  |  |  |
| LCO_2_ | 0.193 | 0.0213 | 9.077 | 0.0003 | 1.0 | 0.4459 | 2.24 | 0.0750 | 0.0034 | 5 |
| MCO_2_ | 0.337 | 0.0247 | 13.647 | < 0.0001 | 1.0 | 0.3496 | 2.86 | 0.0354 | 0.0046 | 5 |
| HCO_2_ | 0.367 | 0.0163 | 22.456 | < 0.0001 | 1.0 | 0.2308 | 4.34 | 0.0075 | 0.0026 | 5 |
| LCO_2_ + MCO_2_ | 0.266 | 0.0371 | 7.172 | < 0.0001 | 1.0 | 0.6092 | 1.64 | 0.1266 | 0.0500 | 12 |
| LCO_2_ + HCO_2_ | 0.281 | 0.0403 | 6.973 | < 0.0001 | 1.0 | 0.6478 | 1.54 | 0.1486 | 0.0674 | 12 |
| MCO_2_ + HCO_2_ | 0.352 | 0.0148 | 23.813 | < 0.0001 | 1.0 | 0.2084 | 4.80 | 0.0004 | 0.0090 | 12 |
| LCO_2_ + MCO_2_ + HCO_2_ | 0.300 | 0.0292 | 10.280 | < 0.0001 | 1.0 | 0.4473 | 2.24 | 0.0376 | 0.0806 | 19 |

An *F*-statistic was calculated as the (RSS/df) of a separate fit, divided by the (RSS/df) of the difference between the separate and combined fit; this was then compared against an *F*-value from an *F*-distribution table using a .05 alpha level.

**Supplementary Table 5. The variance of separated-CO_2_ (e.g. Mid) and combined-CO_2_ (e.g. Low + Mid) growth rate-Feʹ curves, modelled using a P-E equation (**[**Michaelis & Menten, 1913**](#_ENREF_5)**;** [**Platt & Gallegos, 1980**](#_ENREF_7)**); where each curve was normalised to the modelled maximum growth rate (µ_m_) for that CO_2_ and light treatment.**

|  | **µ_m_** | **S.E.** | ***t*-value** | ***p*** | **α** | **S.E.** | ***t*-value** | ***p*** | **RSS** | **df** |
| --- | --- | --- | --- | --- | --- | --- | --- | --- | --- | --- |
| Low Light |  |  |  |  |  |  |  |  |  |  |
| LCO_2_ | 1.0 | 0.0049 | 20.33 | < 0.0001 | 0.0022 | 0.0002 | 9.85 | 0.0006 | 0.0195 | 4 |
| MCO_2_ | 1.0 | 0.0042 | 24.02 | < 0.0001 | 0.0044 | 0.0004 | 9.74 | 0.0006 | 0.0139 | 4 |
| HCO_2_ | 1.0 | 0.0050 | 20.13 | < 0.0001 | 0.0051 | 0.0007 | 7.80 | 0.0015 | 0.0214 | 4 |
| LCO_2_ + MCO_2_ | 1.0 | 0.0056 | 17.66 | < 0.0001 | 0.0031 | 0.0004 | 8.04 | < 0.0001 | 0.1271 | 10 |
| LCO_2_ + HCO_2_ | 1.0 | 0.0069 | 14.04 | < 0.0001 | 0.0035 | 0.0006 | 5.83 | 0.0002 | 0.1968 | 10 |
| MCO_2_ + HCO_2_ | 1.0 | 0.0033 | 30.52 | < 0.0001 | 0.0046 | 0.0004 | 11.64 | < 0.0001 | 0.0444 | 10 |
| LCO_2_ + MCO_2_ + HCO_2_ | 1.0 | 0.0048 | 20.51 | < 0.0001 | 0.0037 | 0.0004 | 8.44 | < 0.0001 | 0.2245 | 16 |
| High Light |  |  |  |  |  |  |  |  |  |  |
| LCO_2_ | 1.0 | 0.0783 | 12.77 | < 0.0001 | 0.0027 | 0.0005 | 5.87 | 0.0020 | 0.0634 | 5 |
| MCO_2_ | 1.0 | 0.0642 | 15.57 | < 0.0001 | 0.0028 | 0.0005 | 5.82 | 0.0021 | 0.0423 | 5 |
| HCO_2_ | 1.0 | 0.0402 | 24.93 | < 0.0001 | 0.0028 | 0.0004 | 7.92 | 0.0005 | 0.0206 | 5 |
| LCO_2_ + MCO_2_ | 1.0 | 0.0460 | 21.73 | < 0.0001 | 0.0027 | 0.0003 | 9.07 | < 0.0001 | 0.1057 | 12 |
| LCO_2_ + HCO_2_ | 1.0 | 0.0389 | 25.73 | < 0.0001 | 0.0028 | 0.0003 | 10.04 | < 0.0001 | 0.0842 | 12 |
| MCO_2_ + HCO_2_ | 1.0 | 0.0338 | 29.58 | < 0.0001 | 0.0028 | 0.0003 | 10.23 | < 0.0001 | 0.0631 | 12 |
| LCO_2_ + MCO_2_ + HCO_2_ | 1.0 | 0.0315 | 31.76 | < 0.0001 | 0.0028 | 0.0002 | 12.22 | < 0.0001 | 0.1265 | 19 |

An *F*-statistic was calculated as the (RSS/df) of a separate fit, divided by the (RSS/df) of the difference between the separate and combined fit; this was then compared against an *F*-value from an *F*-distribution table using a .05 alpha level.

**Supplementary Table 6. The variance of separated-CO_2_ (e.g. Mid) and combined-CO_2_ (e.g. Low + Mid) maximum relative PSII electron transport (rP_m_)-Feʹ curves; where rP_m_ and K_m_^rPm^ were modelled using a Michaelis-Menten equation (**[**Michaelis & Menten, 1913**](#_ENREF_5)**).**

|  | **rP_m_** | **S.E.** | ***t*-value** | ***p*** | **K_m_^rPm^** | **S.E.** | ***t*-value** | ***p*** | **RSS** | **df** |
| --- | --- | --- | --- | --- | --- | --- | --- | --- | --- | --- |
| Low Light |  |  |  |  |  |  |  |  |  |  |
| LCO_2_ | 152.37 | 11.20 | 13.61 | 0.0009 | 21.88 | 35.01 | 0.62 | 0.5763 | 361.4 | 3 |
| MCO_2_ | 153.72 | 26.42 | 5.82 | 0.0101 | 21.73 | 74.75 | 0.29 | 0.7902 | 1860.9 | 3 |
| HCO_2_ | 162.00 | 14.64 | 11.07 | 0.0016 | 35.65 | 24.92 | 1.43 | 0.2480 | 916.4 | 3 |
| LCO_2_ + MCO_2_ | 152.91 | 12.22 | 12.51 | < 0.0001 | 21.36 | 36.27 | 0.59 | 0.5721 | 2226.7 | 8 |
| LCO_2_ + HCO_2_ | 157.26 | 7.35 | 21.38 | < 0.0001 | 31.03 | 15.64 | 1.98 | 0.0825 | 1348.3 | 8 |
| MCO_2_ + HCO_2_ | 158.47 | 11.03 | 14.37 | < 0.0001 | 31.85 | 22.83 | 140 | 0.2004 | 2821.0 | 8 |
| LCO_2_ + MCO_2_ + HCO_2_ | 156.59 | 7.54 | 20.76 | < 0.0001 | 29.73 | 17.33 | 1.72 | 0.1100 | 3219.1 | 13 |
| High Light |  |  |  |  |  |  |  |  |  |  |
| LCO_2_ | 233.41 | 27.04 | 8.63 | 0.0010 | 151.39 | 79.82 | 1.90 | 0.1307 | 2679.0 | 4 |
| MCO_2_ | 222.82 | 6.26 | 35.58 | <0 .0001 | 77.08 | 14.24 | 5.41 | 0.0056 | 317.3 | 4 |
| HCO_2_ | 211.81 | 19.42 | 10.90 | 0.0004 | 17.16 | 13.92 | 1.23 | 0.2851 | 6101.8 | 4 |
| LCO_2_ + MCO_2_ | 225.97 | 12.27 | 18.42 | < 0.0001 | 107.84 | 33.60 | 3.21 | 0.0093 | 3836.2 | 10 |
| LCO_2_ + HCO_2_ | 202.29 | 13.71 | 14.75 | < 0.0001 | 23.34 | 17.56 | 1.33 | 0.2133 | 13597.5 | 10 |
| MCO_2_ + HCO_2_ | 214.92 | 12.12 | 17.74 | < 0.0001 | 38.56 | 18.03 | 2.14 | 0.0582 | 9070.5 | 10 |
| LCO_2_ + MCO_2_ + HCO_2_ | 217.56 | 12.27 | 17.73 | < 0.0001 | 64.32 | 27.41 | 2.35 | 0.0322 | 14647.6 | 16 |

An *F*-statistic was calculated as the (RSS/df) of a separate fit, divided by the (RSS/df) of the difference between the separate and combined fit; this was then compared against an *F*-value from an *F*-distribution table using a .05 alpha level.

**Supplementary Table 7. The variance of separated-CO_2_ (e.g. Mid) and combined-CO_2_ (e.g. Low + Mid) maximum relative PSII electron transport-Feʹ curves, modelled using a Michaelis-Menten equation (**[**Michaelis & Menten, 1913**](#_ENREF_5)**); where each curve was normalised to the modelled maximum relative PSII electron transport (rP_m_) for that CO_2_ and light treatment.**

|  | **rP_m_** | **S.E.** | ***t*-value** | ***p*** | **K_m_^rPm^** | **S.E.** | ***t*-value** | ***p*** | **RSS** | **df** |
| --- | --- | --- | --- | --- | --- | --- | --- | --- | --- | --- |
| Low Light |  |  |  |  |  |  |  |  |  |  |
| LCO_2_ | 1.0 | 0.07 | 13.61 | 0.0009 | 21.88 | 35.01 | 0.62 | 0.5763 | 0.0156 | 3 |
| MCO_2_ | 1.0 | 0.17 | 5.82 | 0.0101 | 21.73 | 74.75 | 0.29 | 0.7902 | 0.0788 | 3 |
| HCO_2_ | 1.0 | 0.09 | 11.07 | 0.0016 | 35.65 | 24.92 | 1.43 | 0.2480 | 0.0349 | 3 |
| LCO_2_ + MCO_2_ | 1.0 | 0.08 | 12.57 | < 0.0001 | 21.78 | 36.15 | 0.60 | 0.5635 | 0.0943 | 8 |
| LCO_2_ + HCO_2_ | 1.0 | 0.05 | 21.65 | < 0.0001 | 35.80 | 16.00 | 2.24 | 0.0556 | 0.0526 | 8 |
| MCO_2_ + HCO_2_ | 1.0 | 0.07 | 14.13 | < 0.0001 | 35.22 | 23.78 | 1.48 | 0.1769 | 0.1163 | 8 |
| LCO_2_ + MCO_2_ + HCO_2_ | 1.0 | 0.05 | 20.56 | < 0.0001 | 34.29 | 18.03 | 1.90 | 0.0795 | 0.1329 | 13 |
| High Light |  |  |  |  |  |  |  |  |  |  |
| LCO_2_ | 1.0 | 0.16 | 8.63 | 0.0010 | 151.39 | 79.82 | 1.90 | 0.1307 | 0.0492 | 4 |
| MCO_2_ | 1.0 | 0.03 | 35.58 | < 0.0001 | 77.08 | 14.24 | 5.41 | 0.0056 | 0.0064 | 4 |
| HCO_2_ | 1.0 | 0.09 | 10.90 | 0.0004 | 17.92 | 13.92 | 1.23 | 0.2851 | 0.1360 | 4 |
| LCO_2_ + MCO_2_ | 1.0 | 0.06 | 17.02 | < 0.0001 | 109.05 | 36.53 | 2.99 | 0.0137 | 0.0859 | 10 |
| LCO_2_ + HCO_2_ | 1.0 | 0.07 | 13.46 | < 0.0001 | 19.48 | 16.87 | 1.16 | 0.2749 | 0.3477 | 10 |
| MCO_2_ + HCO_2_ | 1.0 | 0.06 | 16.67 | < 0.0001 | 37.84 | 18.93 | 2.00 | 0.0735 | 0.2191 | 10 |
| LCO_2_ + MCO_2_ + HCO_2_ | 1.0 | 0.06 | 15.77 | < 0.0001 | 64.47 | 30.87 | 2.09 | 0.0531 | 0.3743 | 16 |

An *F*-statistic was calculated as the (RSS/df) of a separate fit, divided by the (RSS/df) of the difference between the separate and combined fit; this was then compared against an *F*-value from an *F*-distribution table using a .05 alpha level.

**Supplementary Table 8. Mean dissolved inorganic N concentrations (µM) measured from Fe-replete (2 µM EDTA), optimal growth temperature cultures reported in** [**Boatman et al. (2017**](#_ENREF_2)**).**

|  |  | **Low CO_2_** | | **Mid CO_2_** | | **High CO_2_** | |
| --- | --- | --- | --- | --- | --- | --- | --- |
| **Parameters** | **Units** | **LL** | **HL** | **LL** | **HL** | **LL** | **HL** |
| Temperature response |  |  |  |  |  |  |  |
| NH_4_^+^ | µM | 1.66 (0.52) | 1.00 (0.31) | 0.92 (0.20) | 0.90 (0.20) | 1.04 (0.22) | 1.13 (0.24) |
| NO_3_^-^ | µM | 0.11 (0.01) | 0.21 (0.06) | 0.19 (0.04) | 0.21 (0.05) | 0.14 (0.03) | 0.20 (0.04) |
| Light response |  |  |  |  |  |  |  |
| NH_4_^+^ | µM | 0.90 (0.07) | 1.04 (0.30) | 0.69 (0.21) | 0.94 (0.28) | 0.85 (0.25) | 0.94 (0.11) |
| NO_3_^-^ | µM | 0.09 (0.01) | 0.15 (0.04) | 0.09 (0.03) | 0.17 (0.05) | 0.13 (0.04) | 0.18 (0.02) |

*T. erythraeum* IMS101 cultures were fully acclimated to three CO_2_ concentrations (Low CO_2_ = 180 µatm, Mid CO_2_ = 380 µatm and High CO_2_ = 720 µatm), two light intensities (LL = 40 µmol photons m^-2^ s^-1^; HL = 400 µmol photons m^-2^ s^-1^), under Fe replete conditions at optimal temperature (26.2 °C). Nitrate concentrations were measured spectrophotometrically as described by [Collos *et al.* (1999](#_ENREF_4)), and were always less than the detection limit (1 µM) of the method.


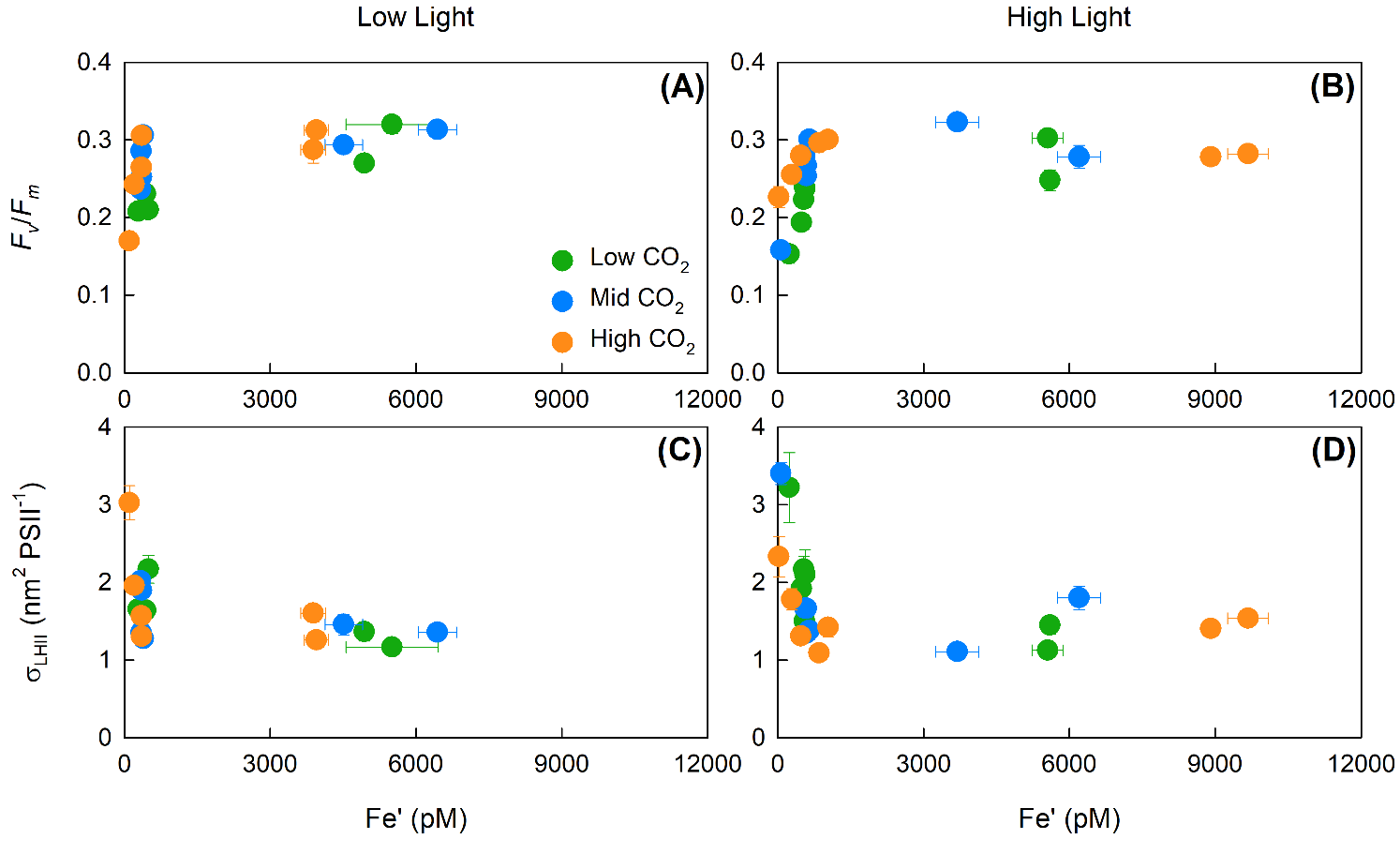
**Supplementary Figure 1. The mean (± S.E.) non-baseline (*F_b_*) corrected photochemical efficiency of PSII in the dark-acclimated state (*F_v_*/*F_m_*) (A,B) and absorption cross section of PSII photochemistry (σ_LHII_ = σ_PII_ / *F_v_*/*F_m_*) for *T. erythraeum* IMS101.** Left-hand panels (A,C) are low light treatments while right-hand panels (B,D) are high light treatments. Cultures were acclimated to three targeted CO_2_ concentrations (Low = 180 µatm (green circles), Mid = 380 µatm (blue circles) and High = 720 µatm (orange circles)), two light intensities (LL = 40 µmol photons m^-2^ s^-1^, HL = 400 µmol photons m^-2^ s^-1^), across a range of Feʹ concentrations (~ 20 - 9600 pM) and at optimal temperature (26 °C).


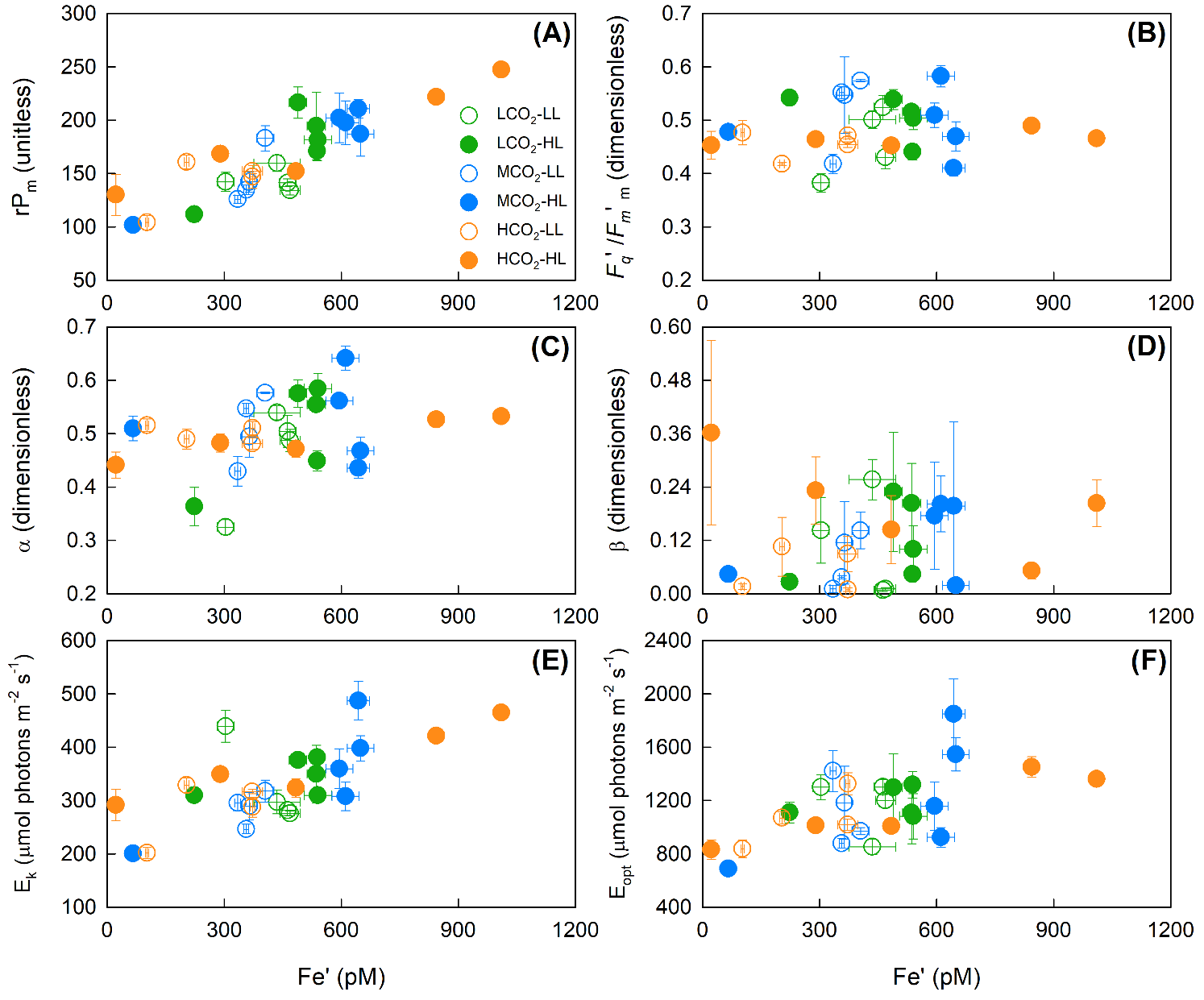
**Supplementary Figure 2.** **The iron-limited mean (± S.E.) maximum relative PSII electron transport rate (rP_m_) (A), photochemical efficiency of PSII in the dark-acclimated state (*F_v_*/*F_m_*) (B), initial slope of the rP-Feʹ curve (α) (C), slope of photoinhibition (β) (D), light-saturated parameter (E_k_) (E) and light intensity at which rP was maximal (E_opt_) (F) for *T. erythraeum* IMS101.** Cultures were acclimated to three targeted CO_2_ concentrations (Low = 180 µatm (green circles), Mid = 380 µatm (blue circles) and High = 720 µatm (orange circles)), two light intensities (LL = 40 µmol photons m^-2^ s^-1^ (open circles), HL = 400 µmol photons m^-2^ s^-1^ (closed circles)), across a range of Feʹ concentrations (~ 20 - 1010 pM), at optimal temperature (26 °C).

**
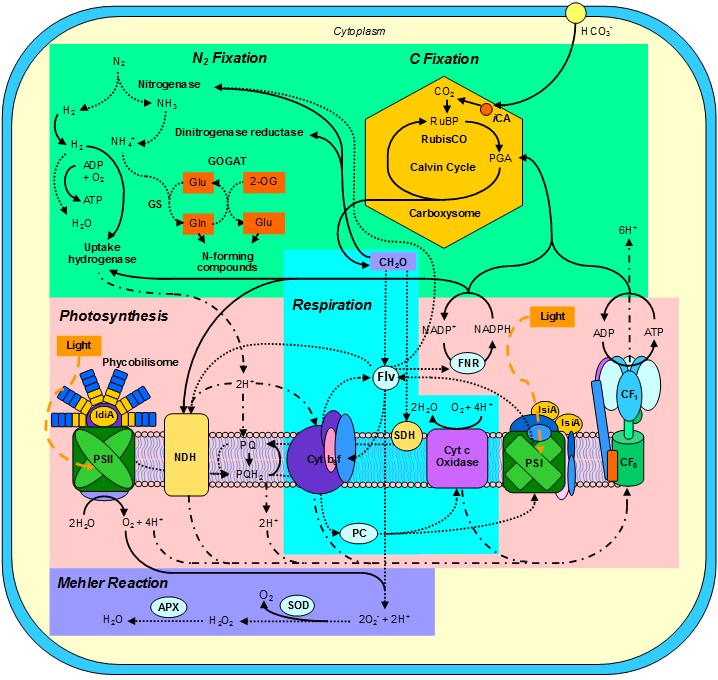
**

**Supplementary Figure 3.** **A metabolic schematic model illustrating the effects of iron limitation on the electron transport among primary components of photosynthesis (pink box), nitrogen and carbon fixation (green box), respiration (blue box) and the Mehler reaction (purple box) in *T. erythraeum* IMS101, as adapted from** [**Shi *et al.* (2007**](#_ENREF_8)**).** Iron deficiency triggers the synthesis of IsiA and IdiA proteins. IsiA forms an additional antenna around PSI ([Bibby *et al.*, 2001](#_ENREF_1); [Boekema *et al.*, 2001](#_ENREF_3)), while IdiA has an implicit role in protecting the acceptor side of PSII to compensate for phycobilisome degradation ([Michel & Pistorius, 2004](#_ENREF_6)). The dashed lines indicate electron flow, and the dash-dot lines indicate proton flow. Note, the over-reduction hypothesis yields an increased production of reactive oxygen species (ROS), potentially compromising the oxygen consumption by the Mehler reaction as several ROS-detoxifying enzymes such as superoxide dismutase (SOD) and ascorbate peroxidase (APX) require Fe as a cofactor. The reduction of N_2_ to NH_4_^+^ requires 2 ATP molecules per electron transfer from dintrogenase reductase to dinitrogenase; therefore, the reduction of N_2_ to NH_4_^+^ requires 16 ATP molecules. Photosystem I transports electrons to flavodoxin (Flv), which can be directed to the following: nitrogenase for N_2_ fixation; Fd oxidoreductase (FNR) for the production of NADPH; Mehler activity for superoxide dismutase (SOD); Cyt*b6f* complex for cyclic electron transport around PSI.


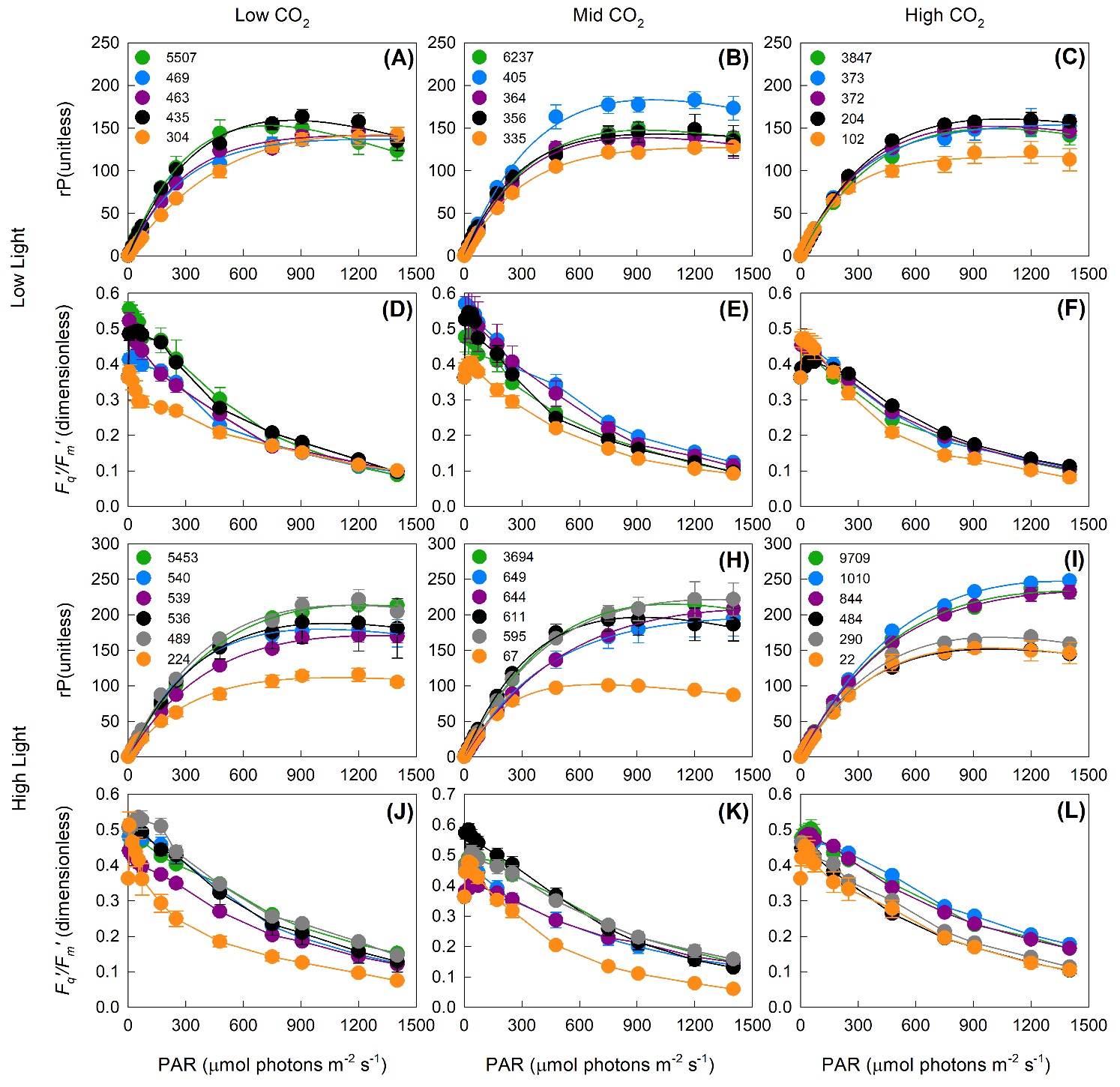
**Supplementary Figure 4.** **The mean (± S.E.) relative PSII electron transport rate (rP) and photochemical efficiency of PSII (*F_q_*ʹ/*F_m_*ʹ) measured during fluorescence light curves (*n* = 3).** *T. erythraeum* was acclimated to low light (40 µmol photons m^-2^ s^-1^) (A-F) and high light (400 µmol photons m^-2^ s^-1^) (G-L) conditions, three targeted CO_2_ concentrations (Low = 180 µatm (Left panels), Mid = 380 µatm (Middle panels) and High = 720 µatm (Right panels)), at optimal temperature (26 °C) and across a range of Feʹ concentrations (~ 102 - 9709 pM).

**References**

**Bibby TS, Nield J, Barber J. 2001. Iron deficiency induces the formation of an antenna ring around trimeric photosystem I in cyanobacteria. *Nature* 412(6848): 743-745.**

**Boatman TG, Lawson T, Geider RJ. 2017. A Key Marine Diazotroph in a Changing Ocean: The Interacting Effects of Temperature, CO_2_ and Light on the Growth of *Trichodesmium erythraeum* IMS101. *PLoS One* 12(1): e0168796.**

**Boekema E, Hifney A, Yakushevska A, Piotrowski M, Keegstra W, Berry S, Michel K-P, Pistorius E, Kruip J. 2001. A giant chlorophyll–protein complex induced by iron deficiency in cyanobacteria. *Nature* 412(6848): 745-748.**

**Collos Y, Mornet F, Sciandra A, Waser N, Larson A, Harrison P. 1999. An optical method for the rapid measurement of micromolar concentrations of nitrate in marine phytoplankton cultures. *Journal of Applied Phycology* 11(2): 179-184.**

**Michaelis L, Menten ML. 1913. Die kinetik der invertinwirkung. *Biochem. z* 49(333-369): 352.**

**Michel KP, Pistorius EK. 2004. Adaptation of the photosynthetic electron transport chain in cyanobacteria to iron deficiency: the function of IdiA and IsiA. *Physiologia Plantarum* 120(1): 36-50.**

**Platt T, Gallegos CL 1980. Modelling primary production. *Primary productivity in the sea*: Springer, 339-362.**

**Shi T, Sun Y, Falkowski PG. 2007. Effects of iron limitation on the expression of metabolic genes in the marine cyanobacterium *Trichodesmium erythraeum* IMS101. *Environmental Microbiology* 9(12): 2945-2956.**
